# Impact of Chronic Risperidone Use on Behavior and Survival of 3xTg-AD Mice Model of Alzheimer’s Disease and Mice With Normal Aging

**DOI:** 10.3389/fphar.2019.01061

**Published:** 2019-09-24

**Authors:** Virginia Torres-Lista, Secundí López-Pousa, Lydia Giménez-Llort

**Affiliations:** ^1^Medical Psychology Unit, Department of Psychiatry and Forensic Medicine, Universitat Autònoma de Barcelona, Cerdanyola del Vallès, Spain; ^2^Institut de Neurociències, Universitat Autònoma de Barcelona, Cerdanyola del Vallès, Spain; ^3^Research Unit and UVaMiD (Memory and Dementia Assessment Unit), Institut d’Assistència Sanitaria, Salt, Spain

**Keywords:** comorbidities, antipsychotics, risk factors, mortality, aging, memory, neuropsychiatric symptoms, social behavior

## Abstract

Psychosis and/or aggression are common problems in dementia, and when severe or persistent, cause considerable patient distress and disability, caregiver stress, and early institutionalization. In 2005, the Food and Drug Administration (FDA) determined that atypical antipsychotics were associated with a significantly greater mortality risk compared to placebo, which prompted the addition of an FDA black-box warning. The American College of Neuropsychopharmacology (ACNP) White Paper, 2008, reviewed this issue and made clinical and research recommendations regarding the use of antipsychotics in dementia patients with psychosis and/or agitation. Increased mortality risk has also been described in cerebrovascular adverse events in elderly users of antipsychotics. In the present work, at the translational level, we used male 3xTg-AD mice (PS1M146V, APPSwe, tauP301L) at advanced stages of the disease reported to have worse survival than females, to study the behavioral effects of a low chronic dose of risperidone (0.1 mg/kg, s.c., 90 days, from 13 to 16 months of age) and its impact on long-term survival, as compared to mice with normal aging. Animals were behaviorally assessed for cognitive and BPSD (behavioral and psychological symptoms of dementia)-like symptoms in naturalistic and experimental conditions (open-field test, T-maze, social interaction, Morris water maze, and marble test) before and after treatment. Weight, basal glucose levels, and IPGTT (i.p. glucose tolerance test) were also recorded. Neophobia in the corner test was used for behavioral monitoring. Survival curves were recorded throughout the experiment until natural death. The benefits of risperidone were limited, both at cognitive and BPSD-like level, and mostly restricted to burying, agitation/vibrating tail, and other social behaviors. However, the work warns about a clear early mortality risk window during the treatment and long-lasting impact on survival. Reduced life expectancy and life span were observed in the 3xTg-AD mice, but total lifespan (36 months) recorded in C57BL/6 × 129Sv counterparts with normal aging was also truncated to 28 months in those with treatment. Sarcopenia at time of death was found in all groups, but was more severe in wild-type animals treated with risperidone. Therefore, the 3xTg-AD mice and their non-transgenic counterparts can be useful to delimitate critical time windows and for studying the physio-pathogenic factors and underlying causal events involved in this topic of considerable public health significance.

## Introduction

Behavioral changes and neuropsychiatric symptoms (NPS), also known as ’’behavioral and psychological symptoms of dementia (BPSD),” may occasionally signal the onset of Alzheimer’s disease (AD) (Reisberg et al., 1987). They are present in 90% of patients as the disease progresses in a neurodegenerative process which is faster and more severe in males in spite of similar incidence of AD among sexes or women showing higher incidence with increasing age (Lapane et al., 2001; Pike, 2017;Rezanni et al., 2019). The prevalence of delusions in patients with AD is between 9% and 63% and that of hallucinations is between 4% and 41%; the rate of aggression is between 11% and 46% and agitation has an even higher prevalence rate among 20–80% (Jeste et al., 2008). Most of these symptoms diminish the quality of life of the patients and, particularly, the psychosis is associated with a rapid cognitive deterioration (reviewed by Cummings, 2000 and Kalman et al., 2008). The cognitive decline characteristic of dementia is also associated with an increase in social vulnerability in humans that sometimes leads to death (Andrew and Rockwood, 2010). Therefore, these neuropsychiatric symptoms present diverse clinical implications in patients, as is the specific case of psychosis, agitation, and aggression, which increase the burden of disease, also resulting a strong cause of distress among the familiar and professional caregivers (Tan et al., 2005).

During the last two decades, the most recent atypical antipsychotic drugs that have been approved by the Food and Drug Administration of the US Department of Health and Human Services (US FDA) are risperidone in 1993, olanzapine in 1996, quetiapine in 1997, ziprasidone in 2001, and aripiprazole in 2002. These atypical antipsychotics have replaced the first-generation antipsychotics such as haloperidol and thioridazine (Schneider et al., 2005 and Jeste et al., 2008). Atypical antipsychotics are used as the first line of pharmacological approach for the treatment of neuropsychiatric symptoms in AD (Ballard et al., 2009). In the present work, we focused on risperidone, one of the most used atypical antipsychotics and co-administered with different types of drugs. At the pharmacological level, risperidone is a selective monoaminergic antagonist, which has a high affinity with serotonergic receptors 5-HT_2_ and dopaminergic D_2_ and binds also to α_1_ adrenergic receptors and with lower affinity to histaminergic H_1_ and α_2_ adrenergic receptors. It has been approved by the FDA for the treatment of schizophrenia (positive and negative symptoms), bipolar disorders, and autism. It is also used in dementia, depression, obsessive-compulsive disorders, personality disorders, and attention deficit hyperactivity disorder (Katz et al., 2007; Shekelle et al., 2007 and Rodriguez-Antona et al., 2009). This drug has a moderate but significant effect on short-term treatment (> 6–12 weeks) for aggression but is limited in long-term therapy, whereas for agitation symptoms, the results are not well established. In addition, there’s an increase in the concerns about adverse outcomes with these treatments, including strokes and death (Ballard et al., 2009).

In April 2005, the FDA issued the following warning for all atypical antipsychotics based on their evaluations: “The FDA informed health professionals and the public about the increased risk of mortality in elderly patients who received atypical antipsychotic drugs for the treatment of dementia-related psychosis. Analyses of 17 placebo-controlled trials involving 5,377 elderly patients with conduct disorders associated with dementia revealed a risk of death in patients treated with the drug between 1.6 and 1.7 times that observed in patients treated with placebo. The mortality rate in the drug-treated patients was approximately 4.5% compared to a rate of approximately 2.6% in the placebo group. Although the causes of death were varied, most of the deaths appeared to be cardiovascular (for example, heart failure, sudden death) or infectious (pneumonia)”. Based on this analysis, the FDA requested manufacturers of the atypical antipsychotic drugs to include information about this risk in the package leaflet of the drug (Jeste et al., 2008). These alerts brought a diversity of opinions in the scientific community. Some authors considered the warnings about atypical antipsychotics as alarming and potentially harmful for patients with dementia, while others were concerned that there was no clear evidence to support a greater benefit in relation to atypical antipsychotics compared to conventional antipsychotics (Trifiro et al., 2009). In a previous work at our UVaMiD neurology unit, we studied the mortality risk in AD patients at advanced ages of the disease who received risperidone therapy, but we could not find any relationship to metabolic syndrome nor history of heart disease (Vilalta-Franch et al., 2008).

This diversity of opinions can be observed in studies of circadian cycle disorders where patients with dementia after a 12-week treatment at a dose of 1.49 mg/day of risperidone reported improvements in total sleep hours, waking hours in bed, insomnia, and other variables related to sleep (Durán et al., 2005). Other studies have indicated that in elderly patients with dementia low doses of risperidone 0.5 and 1 mg were well tolerated and were associated with reductions in BPSD, in particular, agitation, aggression, irritability, delusions, sleep disturbances, anxiety, and phobias. Despite efficacy in the reduction of various adverse symptoms, risperidone and olanzapine should not be used routinely for the treatment of aggression and psychosis in patients with dementia (Onor et al., 2007). Other studies have indicated that risperidone and olanzapine increase the risk of mortality in elderly patients with dementia with an increased risk of the latter over the former (Vilalta-Franch et al., 2008). Also, mortality risks are increased with high doses of atypical antipsychotics and the causes of mortality are cerebrovascular accidents, respiratory diseases, and circulatory disorders (Huybrechts et al., 2012) compared with people who received placebo (Trifiro et al., 2009). Cerebrovascular risks (CVA) are especially observed during the first weeks of treatment; this risk decreases with time and normalizes after 3 months of treatment (Kleijer et al., 2009). Currently, atypical antipsychotics continue to be used under strict supervision and monitoring in some hospitals and/or geriatrics for the BPSD. This is even though older people are more sensitive to their side effects than young and middle-aged adults, in part by the interaction of changes caused by age and pharmacological sensitivity to antipsychotic treatments (Salzman et al., 2008).

Due to the variety of results and the ethical impossibility of conducting new clinical studies in humans, it is important to model the pharmacological responses of antipsychotics in animal models of the disease and to study their effects in the BPSD. At the translational level, the triple transgenic mouse for AD hosts human transgenes PS1/M146V, APPswe, and tau P301L (Oddo et al., 2003a). These rodents uniquely mimic various symptoms of the disease in a temporal and neuroanatomical pattern similar to that observed in humans (Belfiore et al., 2019). The onset of symptoms has been established between 4 and 6 months of age and involves electrophysiological deficits (in LTP, long term potentiation and fEPSP, field excitatory postsynaptic potential) at the hippocampal level, learning and memory problems, cholinergic deficiencies, and emotional disturbances. However, at these ages, the brains of the animals only show presence of intraneuronal immunoreactivity of Aβ (Kitazawa et al., 2005; Giménez-Llort et al., 2007; Oddo et al., 2003a; Oddo et al., 2003b). After 12 months of age, the neuropathological profile finds its parallelism with the advanced stages of the disease in humans, with the characteristic deposits of Aβ and neurofibrillary tangles of tau protein (Oddo et al., 2003a; Oddo et al., 2003b). We have previously shown increased mortality in male 3xTg-AD mice as compared to females, ranging from 33% (Giménez-Llort et al., 2008) to 100% at 15 months of age (García-Mesa et al., 2012) and its relation to impaired neuroimmunoendocrine system (Giménez-Llort et al., 2014). The increased impact of AD (faster, more severe) of male sex has recently also been reported in the human patient (Pike, 2017; Rezanni et al., 2019).

The present study aims to model in 3xTg-AD mice the vulnerability that leads to an increase in mortality observed in patients with AD chronically treated with atypical antipsychotics such as risperidone. Before we can address this modeling, we defined the starting phenotype of the subject of study. To this end, the animals were evaluated in a battery of tests for measuring exploratory activity, anxiety, learning and memory, burying of objects, and social behavior. An assessment of the basal state of glucose and the tolerance response to it when administered intraperitoneally was also made.

## Materials and Methods

### Animals

Homozygous triple-transgenic 3xTg-AD mice harboring human PS1/_M146V_, APP_Swe_, and tau_P301L_ transgenes were genetically engineered at the University of California Irvine, as previously described (Oddo et al., 2003b). Briefly, two independent transgenes (encoding human APPSwe and human tauP301L, both under control of the mouse Thy1.2 regulatory element) were co-injected into single-cell embryos harvested from homozygous mutant PS1M146V knock-in (PS1KI) mice. The PS1 knock-in mice were originally generated as a hybrid C57BL/6 x 129Sv.

Forty-six 12-month-old 3xTg-AD mice (*n* = 23) and C57BL/6 x 129Sv (*n* = 23) wildtype mice (from now, referred as non-transgenic mice, NTg) from litters of a breeding program established in our laboratory at the Medical Psychology Unit, Universitat Autònoma de Barcelona, were used in this study. All the animals were housed three to four per cage and maintained (Makrolon, 35 × 35 × 25 cm) under standard laboratory conditions (12 h light/dark, cycle starting at 8:00h, food and water available *ad libitum*, 22 ± 2ºC, 50–60% humidity). The circadian activity was recorded during one whole light/dark (LD) period, and the rest of the tests from 9:00h to 13:00h.

This study was carried out in accordance with the recommendations of ARRIVE guidelines developed by the NC3Rs (Kilkenny et al., 2010) and the Spanish legislation on “Protection of Animals Used for Experimental and Other Scientific Purposes” and the European Communities Council Directive (2010/63/EU) on this subject. The protocol CEEAH 2481/DMAH 8700 entitled “Risk factors and preventive/therapeutical strategies in Alzheimer’s disease: studies in triple-transgenic 3xTg-AD mice” was approved by Departament de Medi Ambient i Habitatge, Generalitat de Catalunya.

### Experimental Design and Risperidone Treatment

A longitudinal study divided into successive phases including a “before–after treatment” design was performed. The study started at 12 months of age; that in the 3xTg-AD mice has been extensively reported mimicking neuropathological hallmarks of the disease (Belfiore et al., 2019) and that in the NTg mice (C57BL/6 x 129Sv genetic background) corresponds to middle age. The sample of NTg mice was segregated into two groups according to the activity levels exhibited in the corner test (CT) for neophobia and the open-field (OF) test, to be used as controls that will be treated with saline (NTg mice with low motor activity) or risperidone (NTg mice with high motor activity), respectively.

Risperidone was used at a dose of 0.1 mg/kg equivalent to that administered in patients with AD and used in most experimental work performed in rodents (Bruins Slot et al., 2005). The chronic administration, subcutaneous for 3 months from 13 to 16 months of age, rotated three injection sites (the neck and the two flanks).

First, we characterized the basal phenotype (*phase 1*, weeks 1–6, phenotype “before treatment”; animals at 12 months of age). As in the case of geriatric patients, the treatment regimen was initiated with a lower dose of 0.05 mg/kg (*phase 2*, low dose and follow-up tests, week 7; animals at 13 months of age). After 7 days, the final dose of 0.1 mg/kg was started and behavioral effects assessed (*phase 3*, treatment and behavioral effects “after treatment”, weeks 8–16; animals until 15 months of age). Thereafter, treatment followed without behavior and completed the total period of 3 months of subcutaneous treatment (*phase 4*, only treatment, until 16 months of age). From that moment and until the end of their days, the variables of weight and survival were recorded continuously with a weekly or daily cadence, respectively (*phase 5*, from 16 to 36 months of age).

Four experimental groups were studied and are plotted in the before/after graphs of the figures as follows: NTg mice (s) (NTg mice that will receive or have received saline, *n* = 12), NTg mice (r) (NTg mice that will receive or have received risperidone, *n* = 11), 3xTg-AD mice (s) (3xTg-AD mice that will receive or have received saline, *n* = 12), and 3xTg-AD (r) (3xTg-AD mice that will receive or have received risperidone, *n* = 11).

### Behavioral Assessments

Behavioral assessment consisted in a battery of naturalistic and experimental conditions (Giménez-Llort et al., 2007). Neophobia in the CT was used for behavioral monitoring through the treatment.

### Corner Test (CT)

Animals were individually placed in the center of a clean standard home cage, filled with wood shave bedding. Number of corners visited were recorded during 30 s (Belzung and Le Pape, 1994). Latency to realize the first rearing, and the number of rearings were also registered (Giménez-Llort et al., 2007).

### Open Field Test (OF)

Immediately after the CT, mice were placed in the center of an open field (homemade woodwork, white box, 50 × 50 × 20 cm) and observed for 5 min (Hall and Ballachey, 1932). The ethogram, described by the temporal profile of the following sequence of behavioral events, was recorded: duration of freezing behavior, latency to leave the central square and that of entering the peripheral ring, as well as latency and total duration of self-grooming behavior. Horizontal (crossings of 10 × 10 cm squares) and vertical (rearings with a wall support) locomotor activities were also measured. Bizarre behaviors observed in this test were also measured according to the previous reported criterion (Baeta-Corral and Giménez-Llort, 2014). During the tests, defecation boli and urination were also recorded as measures of individual differences in emotionality (Hall, 1934).

### T-Maze (TM)

Working memory was assessed by means of a spontaneous alternation task (Douglas, 1966) in a black TM. The apparatus consisted of a woodwork, three arms of 30 × 5 × 20 cm connected by a 5 × 5 × 20 cm intersection. The animal was placed inside the “vertical” arm of the maze with its head facing the end wall, and it was allowed to explore the maze during a maximum of 3 min. Freezing behavior (latency to move), the latency to reach the intersection, the total time invested to explore the three arms of the maze, and the number of errors (revisiting an arm) were recorded. Defecation boli and urination were also noted.

### Social Interaction Test (SIT)

Behavioral signatures of social dysfunction in 3xTg-AD mice were assessed by means of the SIT (File and Hyde, 1978) as recently described (Torres-Lista and Giménez-Llort, 2019). A dyad of two unfamiliar mice of the same genotype and sex were introduced in a standard home cage and video recorded for 5 min. Behaviors were classified into social (social investigation, aggression, vibrant tail) and non-social (exploring, digging, self-grooming) interactions. We also scored the total number of episodes and their total duration.

### Morris Water Maze (MWM)

A 5-day place learning task for short- and long-term spatial reference memory (four trial sessions per day, with trials spaced 30 min apart) was followed 2 h 30 min later by a probe trial (removal of the platform) for short-term memory in the MWM (Morris, 1981; Morris, 1984). Mice were trained to locate a hidden platform (7-cm diameter, 1 cm below the water surface) in a circular pool for mice (Intex Recreation Corp., Long Beach, CA, United States; 91-cm diameter, 40-cm height, 25°C opaque water), located in a completely black painted 6-m^2^ test room. Mice that failed to find the platform within 60 s were placed on it for 10 s, the same period as was allowed for the successful animals. White geometric figures, one hung on each wall of the room, were used as external visual clues. Behavior was evaluated by direct observation and analysis of videotape-recorded images. Variables of time (escape latency) and quadrant preference and entries were analyzed in all the trials of the tasks. The escape latency was readily measured with a stopwatch by an observer unaware of the animal’s genotype and confirmed during the subsequent video-tracking analysis. In the probe trial, the time spent and number of entries in each of the four quadrants were also measured retrospectively by means of the automated video-tracking analysis.

### Marble Burying Test (MB)

The procedure for MB was adopted with minor modifications from that originally described by Broekkamp et al. (1986). Mice were placed individually in a standard home cage containing six glass marbles (1 × 1 × 1 cm) evenly spaced making a triangle (three rows of three, two, and one marble per row only in the left area of the cage) on a 5-cm-thick layer of sawdust. The mice were left in the cage with marbles for a 30-min period after which the test was terminated by removing the mice and counting the number of marbles: intact (untouched), rotated or at least half buried by sawdust, and buried (completely hidden) as previously described (Torres-Lista et al., 2015)

### Body Weight (BW) and Basal Glucose Levels (G)

Throughout the experimental process, evaluation of weight and survival was continuously monitored until the natural end of the life of the animals. The blood samples were taken from an incision made at the tip of the tail.

### Survival Curve

The survival curves were obtained with the percentage of animals that were maintained throughout the experimental procedure.

### Statistics

Statistical analysis were performed using SPSS 17.0 software. The results are expressed as means ± SEM or percentage. A 2×2 factorial design with multivariate general lineal model analysis evaluated genotype (G) and treatment (T) effects, followed by *post hoc* Tukey B test. In the Morris water maze, the factor ‘day (D)’ was included when appropiate. Student’s *t*-test was used to compare two independent groups. The comparisons for related samples were made with the paired *t*-test. Survival curve was analyzed with Kaplan-Meier test. The correlations between survival and the different variables studied were evaluated with the Pearson’s correlation. In all the tests, P < 0.05 was considered statistically significant.

### Results

**Figure 1:**
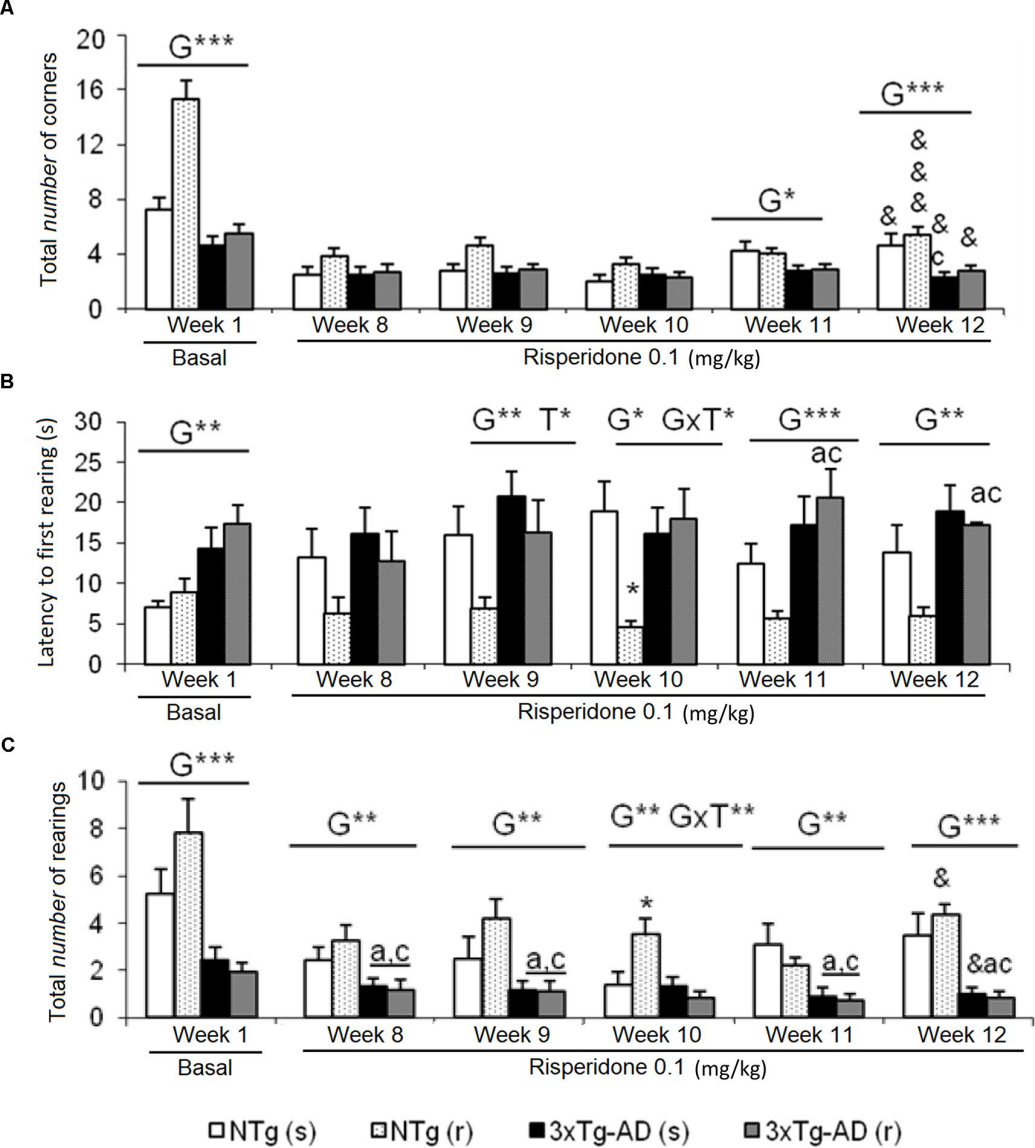
Effects of chronic risperidone assessed in the corner test. Results in the corner test before (basal) and through different weeks of treatment. **(A)** Number of corners, **(B)** latency of first rearing, and **(C)** number of rearings. ANOVA 2×2, genotype effect (G), genotype × treatment interaction (G×T), ***p < 0.001, **p < 0.01, and *p < 0.05. *Post hoc* Tukey B test *p < 0.05 *vs*. all other experimental groups; ^a^*p* < 0.05 *vs*. different genotype but the same treatment; and ^c^*p* < 0.05 *vs*. different genotype and different treatment. *t -test* for paired data, treatment effect: treatment with a dose of 0.1 mg/kg *vs*. no treatment, ^&&&^*p* < 0.001 and ^&^*p* < 0.05.

**Figure 2:**
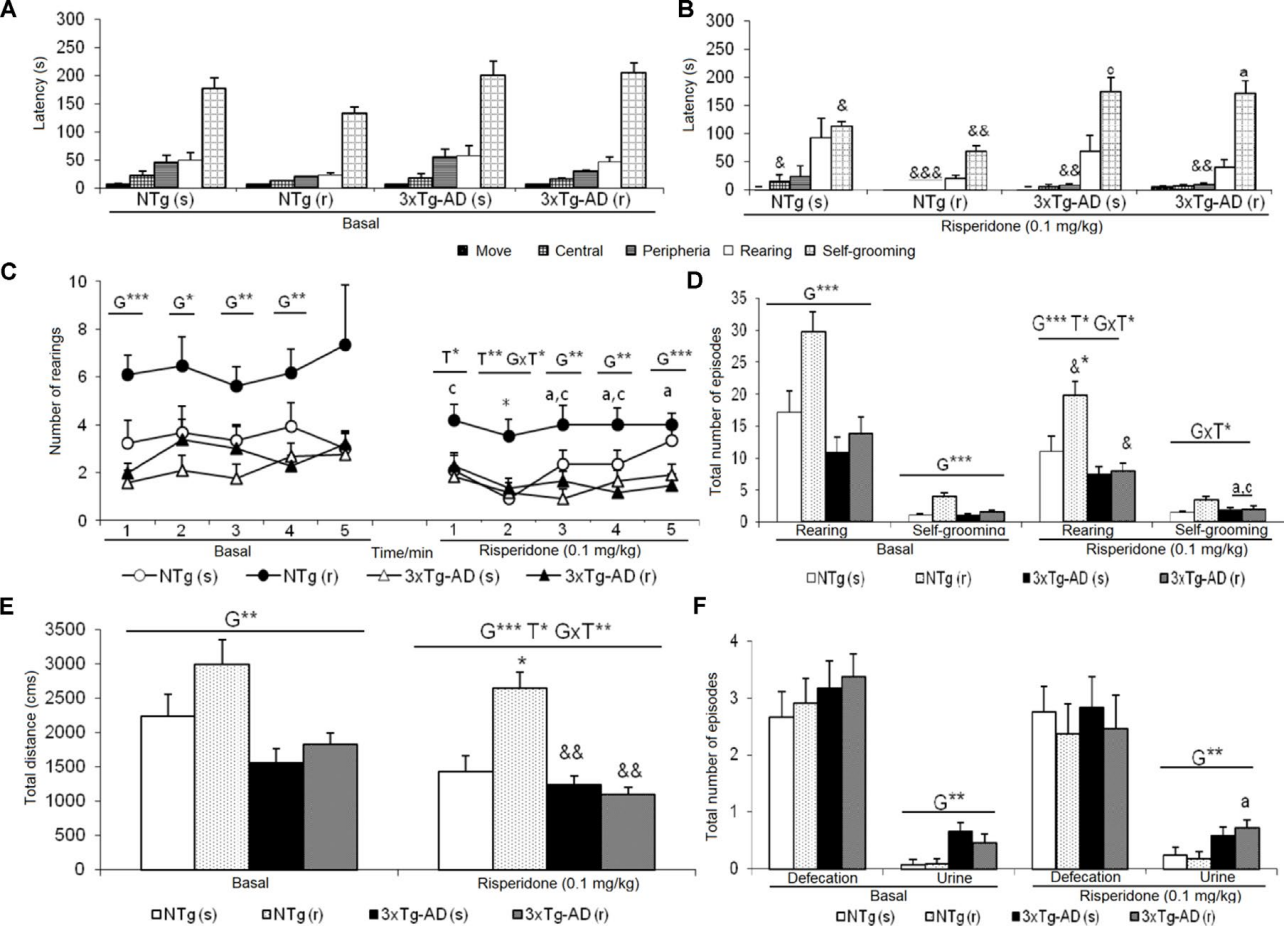
Effects of chronic risperidone assessed in the open field test. Results in the open field test before (basal) and during treatment. **(A)** Latencies before treatment; **(B)** latencies after treatment; **(C)** number of rearings; **(D)** total number of episodes of rearing and self-grooming; **(E)** total distance covered; **(F)** total number of episodes of defecation and urine. ANOVA 2×2, genotype effect (G), treatment (T) and genotype × treatment interaction (G×T), ***p < 0.001, **p < 0.01, and *p < 0.05. *Post hoc* Tukey B test ^a^ *p* < 0.05 *vs*. different genotype but the same treatment and ^c^ *p* < 0.05 *vs*. different genotype and different treatment. Effect *t*-test for paired data, treatment effect: treatment with a dose of 0.1 mg/kg *vs*. before treatment ^&&&^ *p* < 0.001, ^&&^ *p* < 0.01, and ^&^ *p* < 0.05.

**Figure 3:**
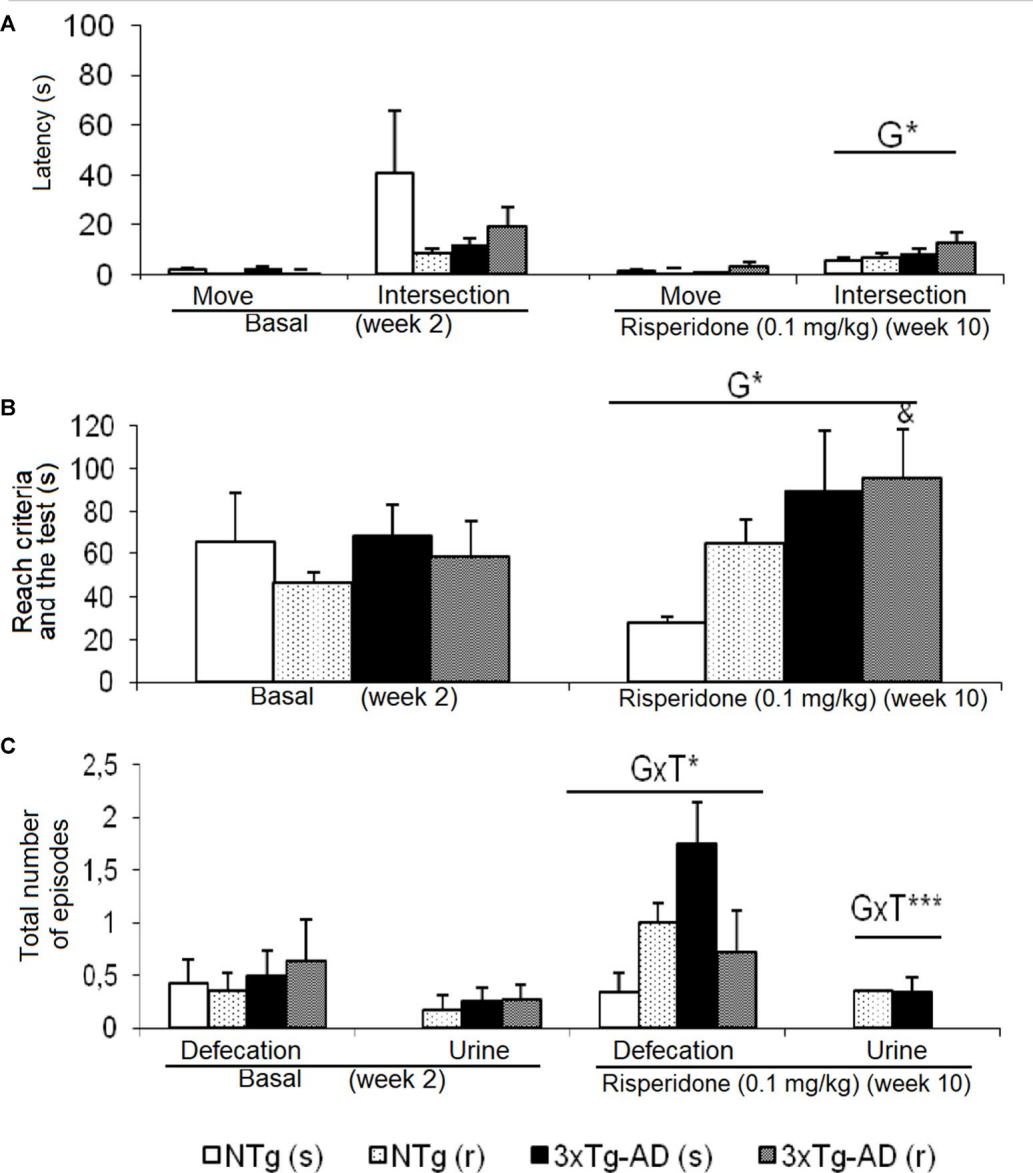
Effects of chronic risperidone assessed in the T-Maze before (basal) and during the treatment. **(A)** Latencies (s), **(B)** time to complete the maze (s), and **(C)** total number of defecations and urine. ANOVA 2×2, genotype effect (G), genotype × treatment (GxT), ***p < 0.001 and *p < 0.05. *Post hoc* Tukey B test ^a^ *p* < 0.05 *vs*. different genotype but the same treatment and ^c^ *p* < 0.05 *vs*. different genotype and different treatment. *t*-Test effect for paired data, treatment effect: treatment with a dose of 0.1 mg/kg vs. no treatment, ^&^ *p* < 0.05.

**Figure 4:**
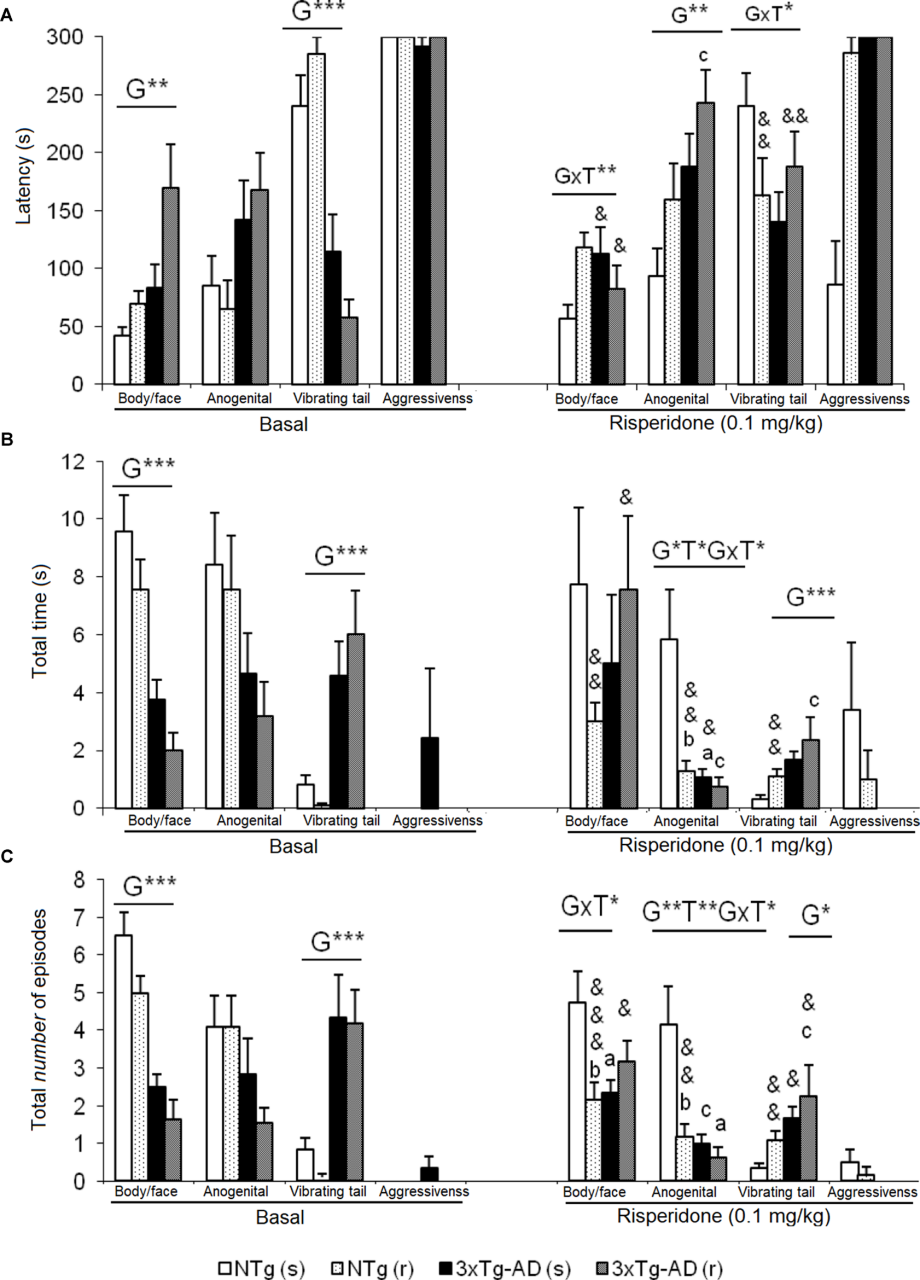
Effects of chronic risperidone in the social behaviors assessed in the social interaction test before (basal) and during the treatment. **(A)** Latencies (s). ANOVA 2×2, genotype effect (G), genotype × treatment interaction (G×T), **p < 0.01 and *p < 0.05. *Post hoc* Tukey B test *p < 0.05. *vs*. all other experimental groups; ^a^ *p* < 0.05 *vs*. different genotype but the same treatment and ^c^ *p* < 0.05 vs. different genotype and different treatment. *t*-test effect for paired data, treatment effect (T): treatment with a dose of 0.1 mg/kg *vs*. no treatment, ^&&^ *p* < 0.01 and ^&^ *p* < 0.05. **(B)** Total time and **(C)** total number of episodes. ANOVA 2×2, genotype effect (G) treatment effect (T) and genotype × treatment interaction effect (G×T), ***p<0.001 **p < 0.01 and *p < 0.05. *Post hoc* Tukey B test *p < 0.05. *vs*. all other experimental groups; at *p* < 0.05 *vs*. different genotype but the same treatment; ^b^ *p* < 0.05 *vs*. different treatment but the same genotype and ^c^ *p* < 0.05 *vs*. different genotype and different treatment. *t*-test effect for paired data, treatment effect: treatment with a dose of 0.1 mg/kg *vs*. no treatment, ^&&&^ *p* < 0.001, ^&&^ *p* < 0.01, and ^&^ *p* < 0.05.

**Figure 5:**
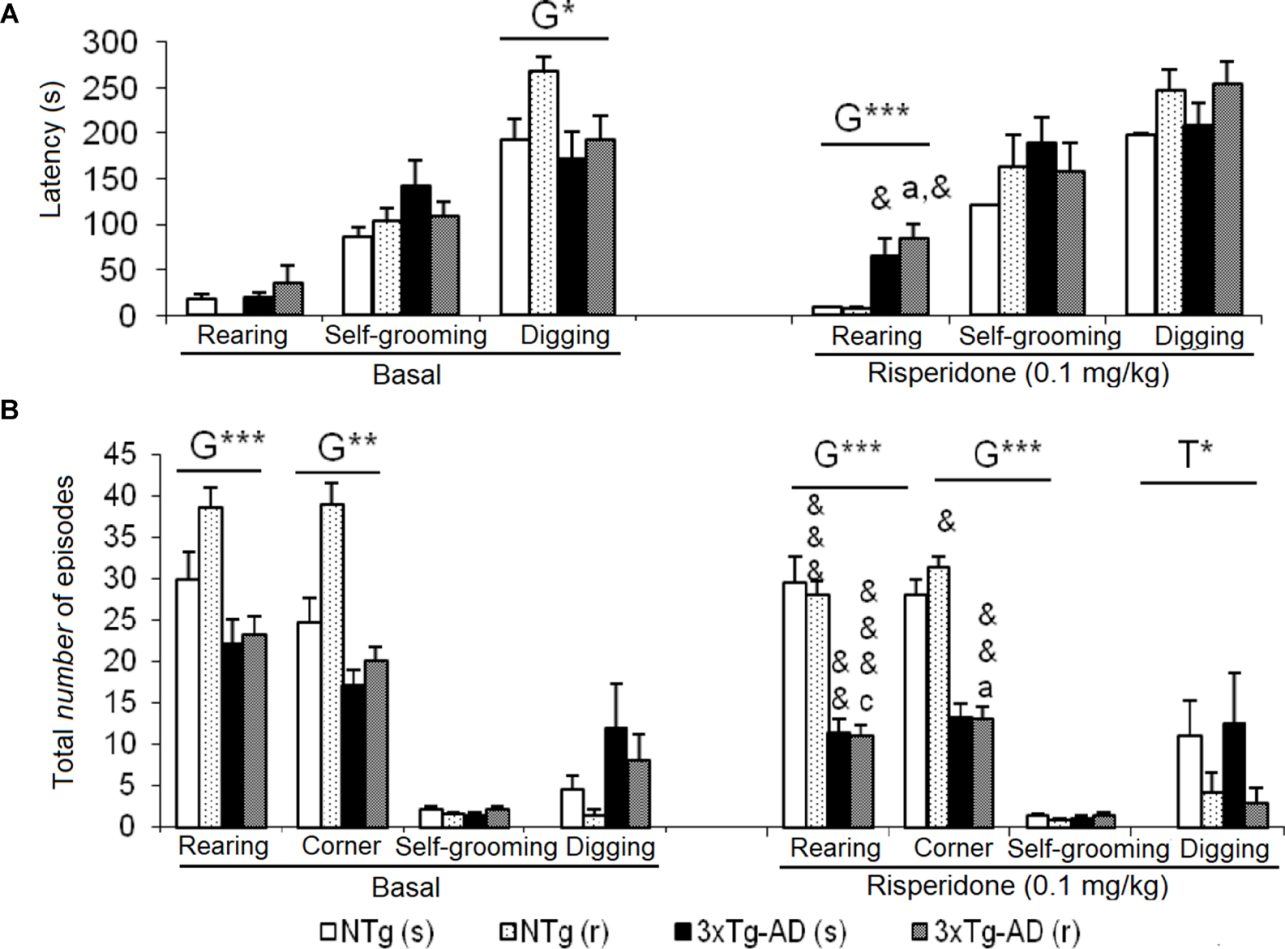
Effects of chronic risperidone in the non-social behaviors assessed in the social interaction test before (basal) and during the treatment. **(A)** Latencies (s) and **(B)** total number. ANOVA 2×2, genotype effect (G), treatment (T), ***p < 0.001 and *p < 0.05. *Post hoc* Tukey B test ^a^ *p* < 0.05 *vs*. different genotype but the same treatment and ^c^ *p* < 0.05 *vs*. different genotype and different treatment. Effect *t*-test for paired data, treatment effect: treatment with a dose of 0.1 mg/kg *vs*. untreated ^&&&^ *p* < 0.001, ^&&^ *p* < 0.01, and ^&^ *p* < 0.05.

**Figure 6:**
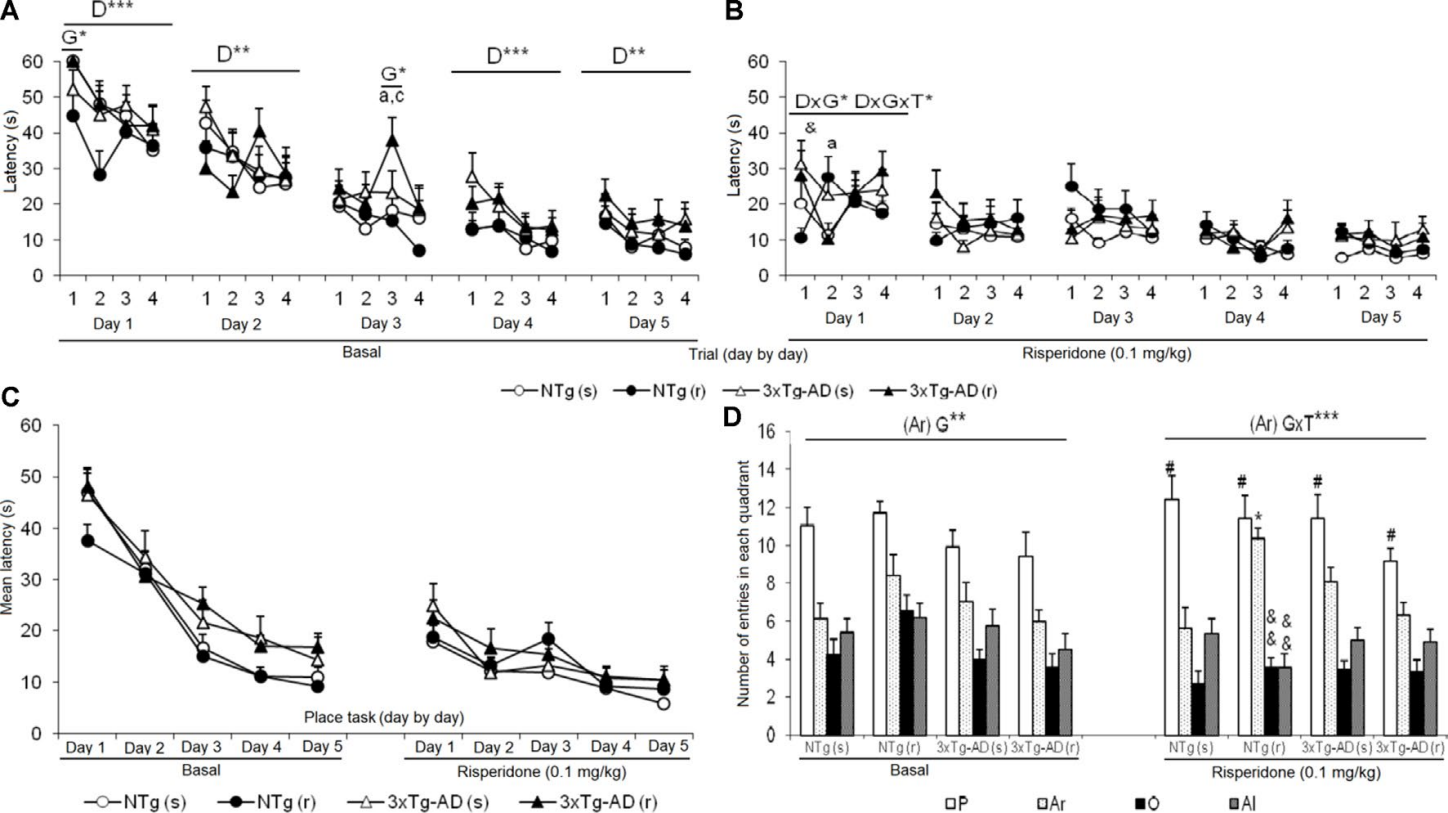
Effects of chronic risperidone in the Morris water maze before (basal) and during the treatment genotype effect (G). Latencies (s) evolution before **(A)** and after **(B)** the treatment, evolution of learning and memory, trial by trial for each of the 5 days. Repeated measures ANOVA, 2×2×5, day effect (D), day × genotype effect (DxG), and interaction effect (D×G×T), *p < 0.05, **p < 0.01, and ***p < 0.001. *Post hoc* Tukey B test ^a^ *p* < 0.05 vs. different genotype but the same treatment and ^c^ *p* < 0.05 *vs*. different genotype and different treatment. *t*-Test for paired data, treatment effect: trial 1 of day 1 with treatment *vs*. trial 4 of day 5 without treatment, ^&^ *p* < 0.05. **(C)** Latency (s), evolution of learning and memory day by day. Repeated measures ANOVA 2×2×5, ns and *post- hoc* Tukey B test, ns **(D)** total number of entries in the quadrant: platform (P), right (Ar), opposite (O), and left (Al). ANOVA 2×2, genotype × treatment interaction effect (G×T), ***p < 0.001. *t*-Test for paired data, treatment effect: treatment with a dose of 0.1 mg/kg vs no treatment, ^&&^ *p* < 0.01 and ^&^ *p* < 0.05. ANOVA and *post hoc* Tukey B test interaction of the three levels #p < 0.001.

**Figure 7:**
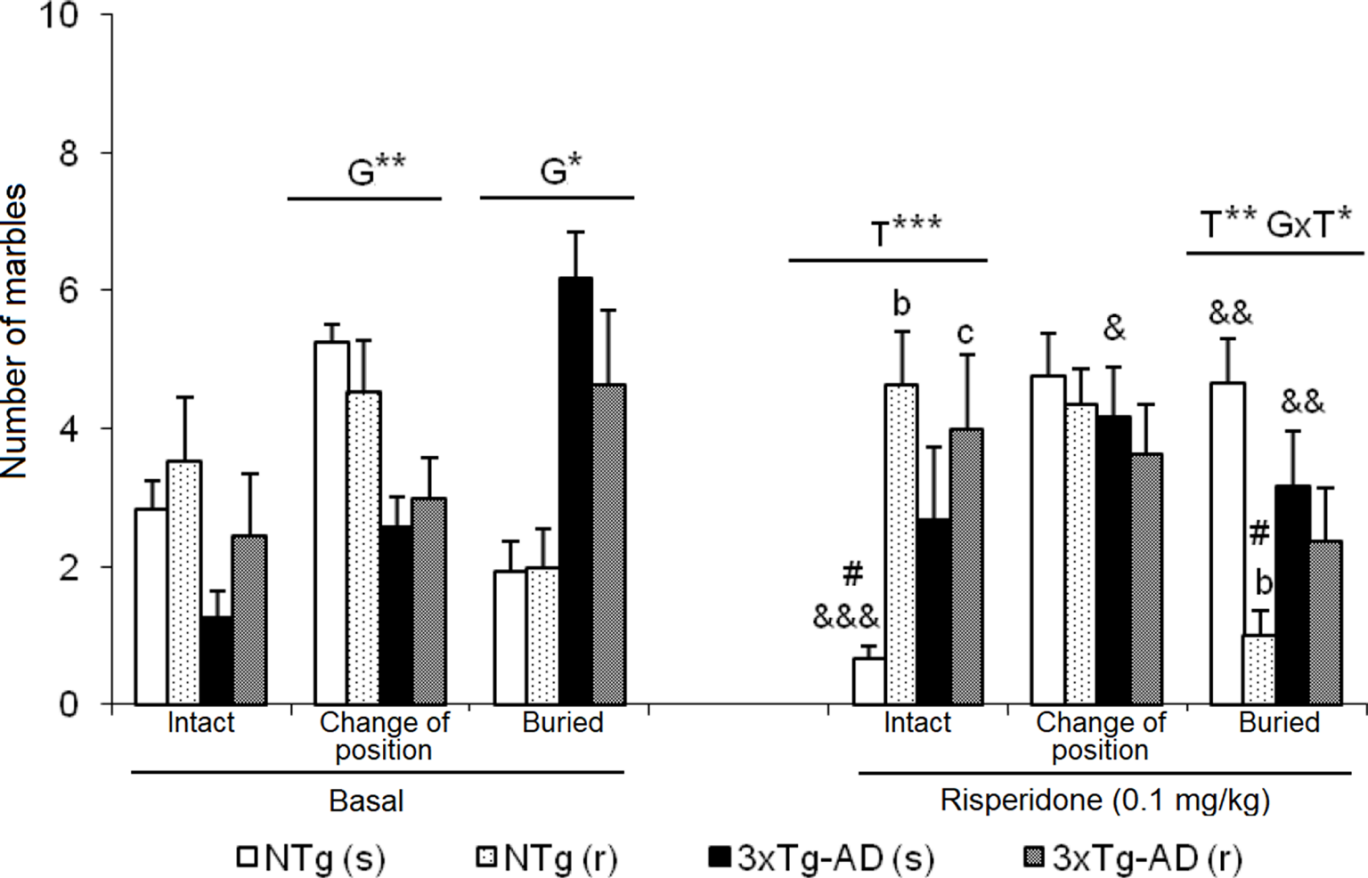
Effects of chronic risperidone in the marble burying test before (basal) and during the treatment. Number of marbles. *t*-Test for paired data, treatment effect: treatment with a dose of 0.1 mg/kg vs no treatment, ^&&&^ *p* < 0.001, ^&&^ *p* < 0.01, and ^&^ *p* < 0.05. ANOVA and *post hoc* Tukey B test interaction of the three levels ^#^ *p* < 0.001. ANOVA 2×2, (T), treatment effect and (G×T), genotype × treatment interaction, ***p < 0.001, **p < 0.01, and *p < 0.05. *Post hoc* Tukey B test ^b^ *p* < 0.05 vs. different treatment but the same genotype and ^c^ *p* < 0.05 vs. different genotype and different treatment.

**Figure 8:**
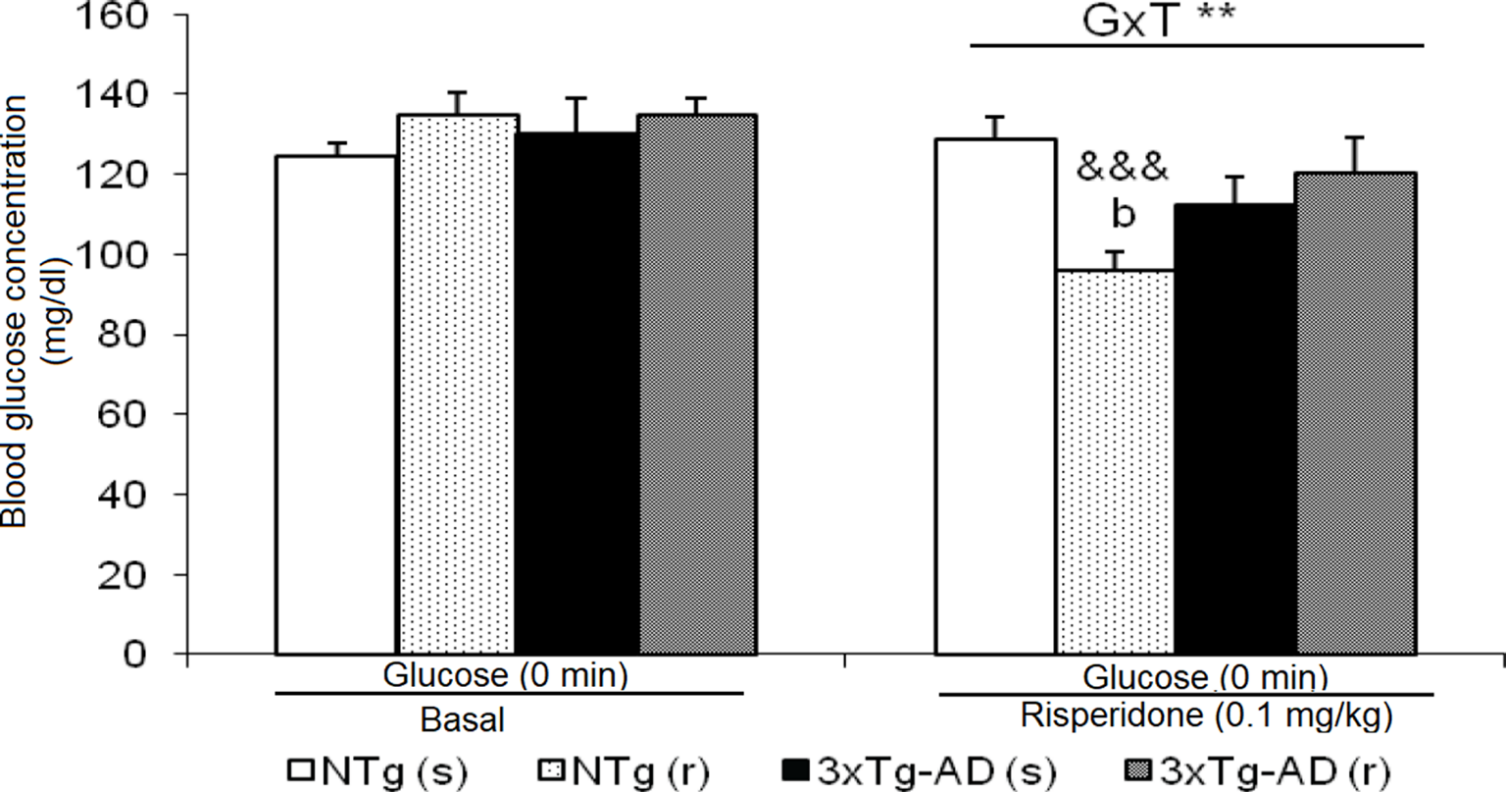
Effects of chronic risperidone in basal state of glucose before and during the treatment. *t*-Test for paired data, treatment effect: treatment with a dose of 0.1 mg/kg vs no treatment, ^&&&^ *p* < 0.001. ANOVA 2×2, interaction effect genotype × treatment (G×T), **p < 0.01. *Post hoc* Tukey B test ^b^ *p* < 0.05 vs. different treatment but the same genotype.

**Figure 9:**
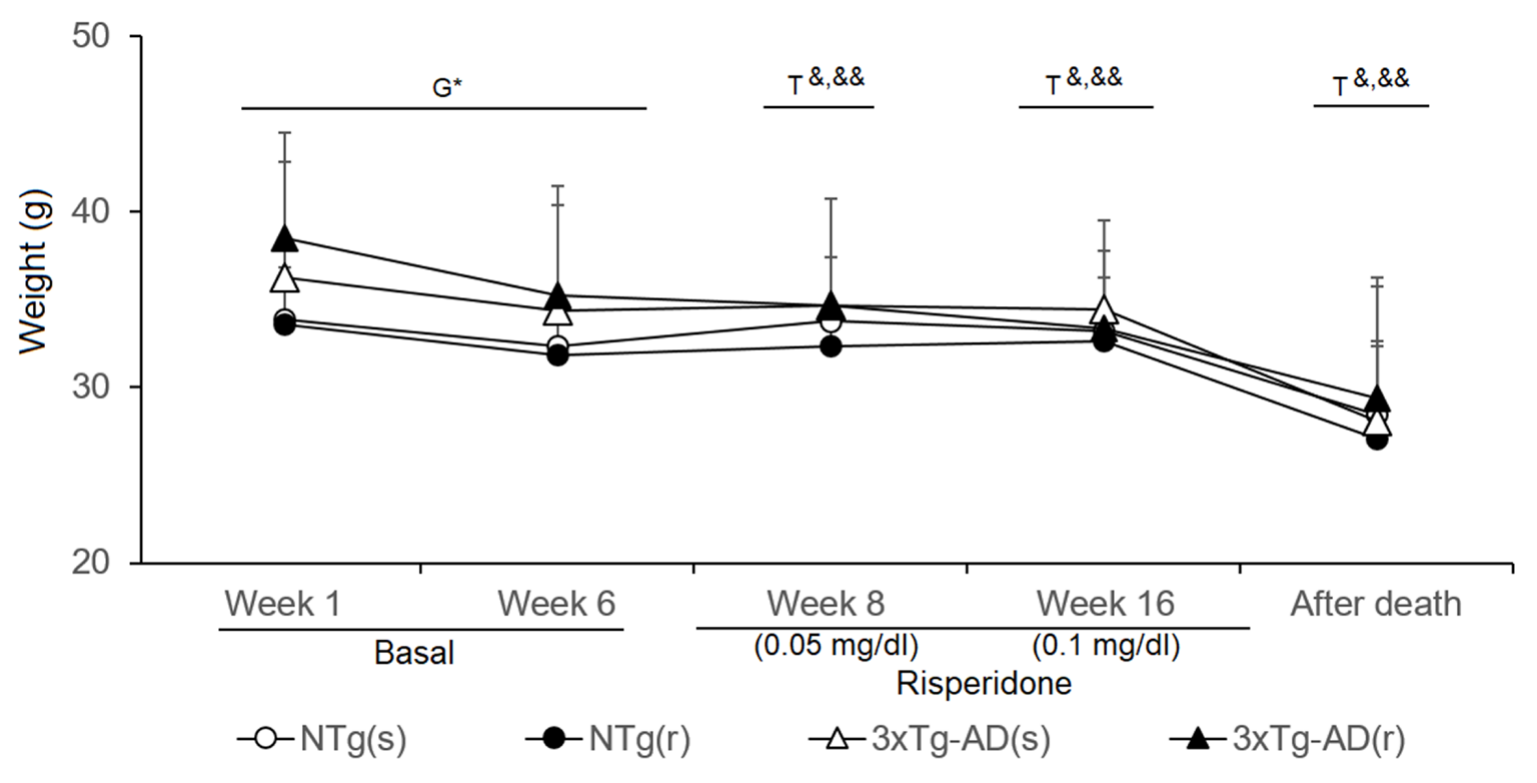
Effects of chronic risperidone in the body weights of the animals before (basal), during the treatment, and immediately after death Phase 1 Basal. Student’s *t*-test; *p < 0.05. Phase 2. Start of treatment with risperidone at a dose of 0.05 mg/kg. Weights. *t*-Test for paired data, treatment effect: treatment with a dose of 0. 05 mg/kg vs untreated, ^&&^ *p* < 0.01 ^&^ *p* < 0.05 for Phase 3. Treatment with risperidone at a dose of 0.1 mg/kg. *t*-Test for paired data, treatment effect: treatment with a dose of 0. 1 ml/kg vs untreated ^&&^ *p* < 0.01 ^&^ *p* < 0.05 Phase 4. Immediately after death vs. the weights at the beginning of the longitudinal study. *t*-Test for paired data ^&&^ *p* < 0.01, and ^&^ *p* < 0.05.

**Figure 10:**
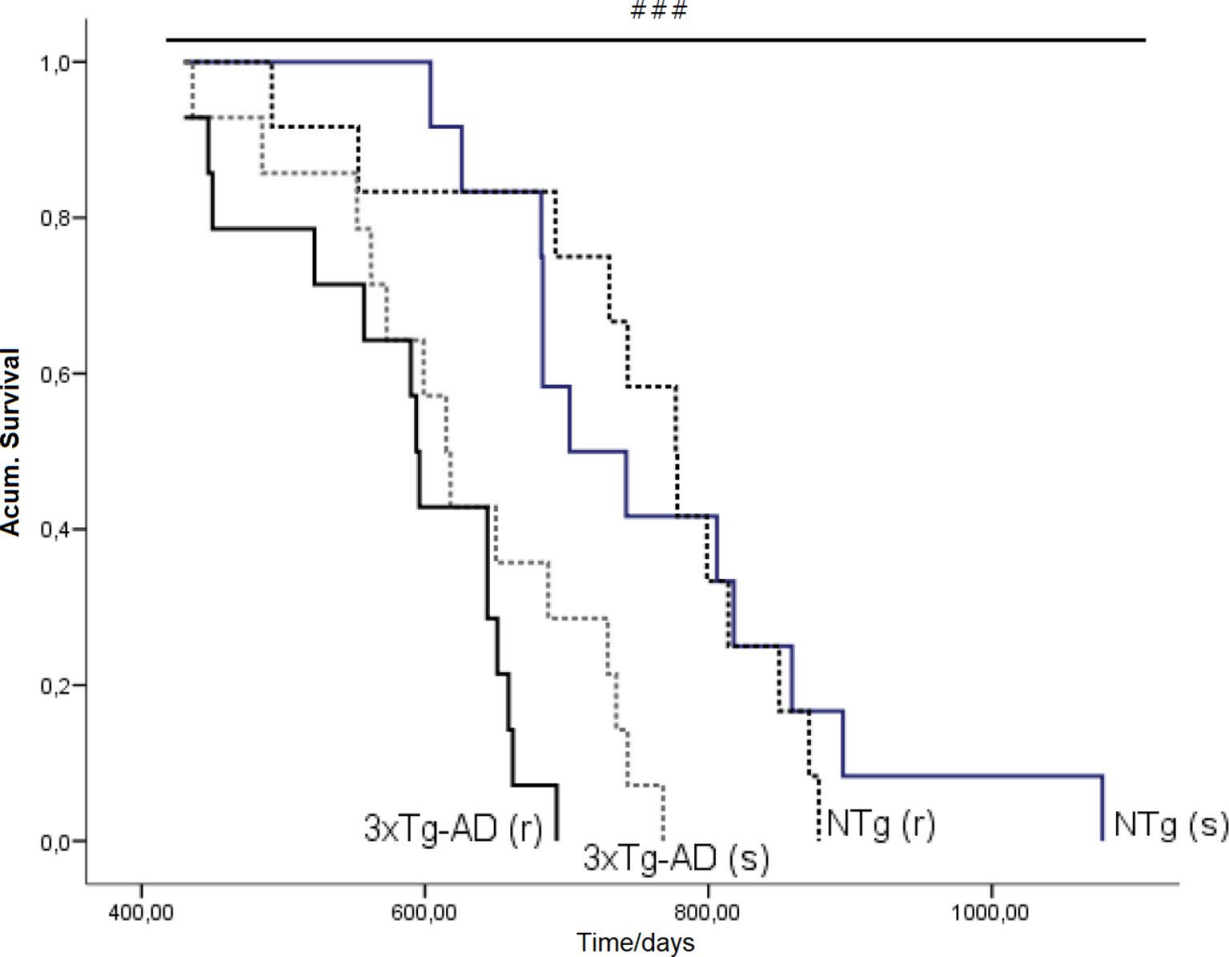
Effects of chronic risperidone in the total mean survival. Mean ± SEM. Survival curves based on the age of the animals (in days). Each line represents the cumulative survival of the animals of each experimental group [NTg (s) and 3xTg-AD (s)] Log-rank test *p *< 0.001. Survival curve based on the age of the animals (in days). Each line represents the cumulative survival of the animals of each experimental group [NTg (s), NTg (r), 3xTg-AD (s) and 3xTg-AD (r)]. Log-rank-test ^###^ *p* < 0.001.

**Figure 11:**
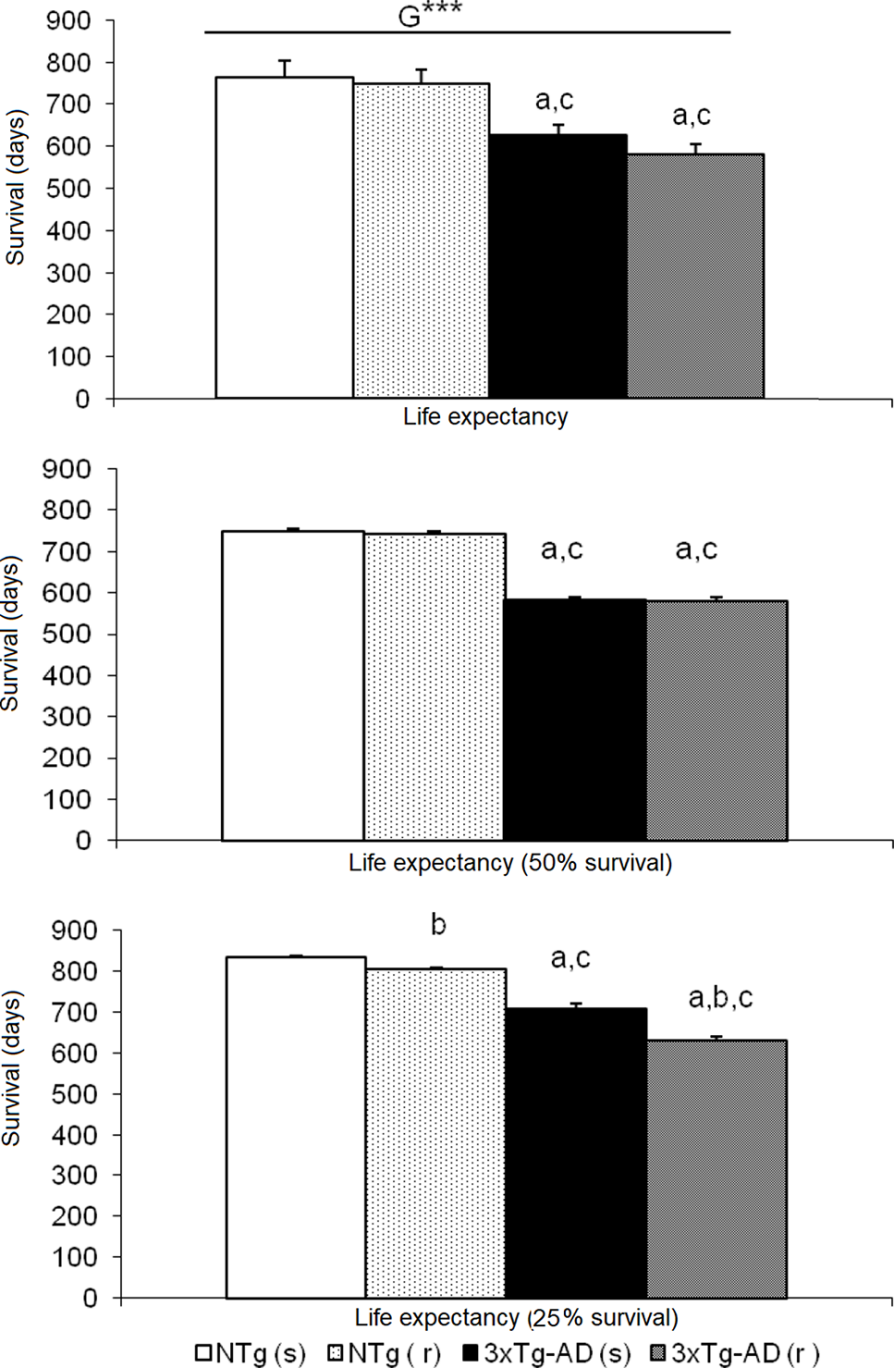
Effects of chronic risperidone in the total mean survival. Mean ± SEM. ANOVA and *post hoc* Tukey B test ***p<0.001, ^a^ *p* < 0.05 vs. same treatment but different genotype, ^b^ *p* < 0.05 vs. different treatment but the same genotype and ^c^ *p* < 0.05 vs. different treatment different genotype.

**Figure 12:**
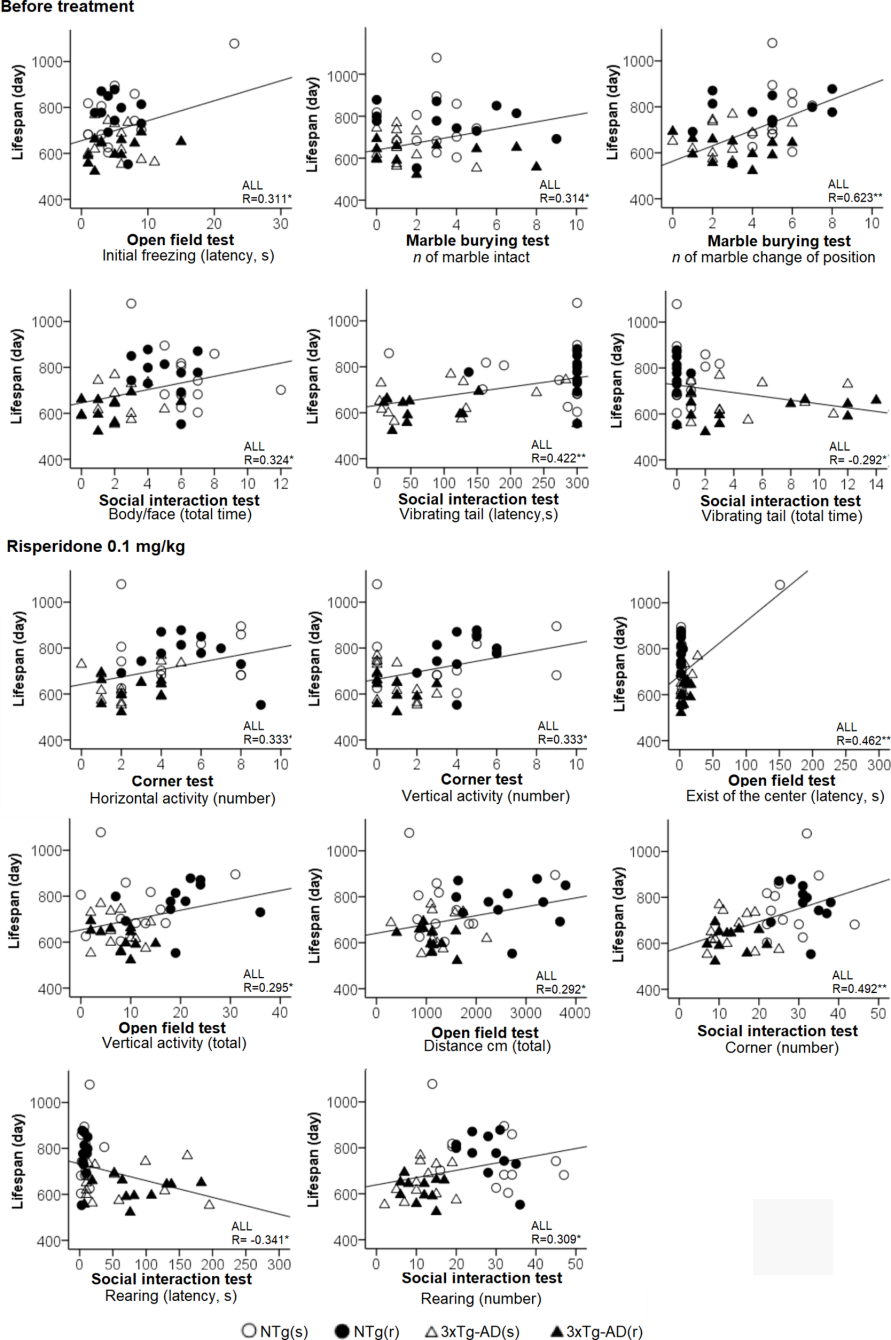
Behavioral correlates for lifespan considering all the sample of animals. Pearson’s correlations between behavioral variables and lifespan: *p < 0.05, **p < 0.01.

We confirmed that the sample of 3xTg-AD mice studied exhibited cognitive deficits in the MWM, mimicking the cognitive hallmark of AD. However, only data of animals that could be included in the “before–after” analysis were considered (animals dying in phases 1, 2, and 3 were excluded). Statistics of genotype effects for the different behavioral tests and variables studied in 3xTg-AD and NTg mice at 12 months of age (week 1, basal, but without the segregation for the treatment they will receive) are cited in the text and depicted in **Supplementary Table S1**. Behavioral correlates with lifespan in animals treated with saline or risperidone are also indicated. **Figures 1–12** depict the effects of chronic risperidone on these behaviors, weight, and survival curves of animals. Finally, **Table 1** details the behavioral correlates with lifespan in male NTg and 3xTg-AD mice chronically treated with saline or risperidone.

**Table 1:** Behavioral correlates with lifespan in male NTg and 3xTg-AD mice chronically treated with saline or risperidone.

*Behavioral test and variable*	Lifespan (days)			
	NTg(s)	NTg(r)	3xTg-AD(s)	3xTg-AD(r)
***Before treatment.***				
*Corner Test *				
Vertical activity (latency, s)	0.684*	*n.s.*	*n.s.*	*n.s.*
Vertical activity (number)	-0.614*	*n.s.*	*n.s.*	*n.s.*
*Open field test*				
Initial movement (latency of freezing, s)	0.715**	*n.s.*	*n.s.*	*n.s.*
Exist of the center (latency, s)	0.63**	*n.s.*	*n.s.*	*n.s.*
Vertical activity (latency, s)	*n.s.*	*n.s.*	*n.s.*	-0.606*
Self-grooming (latency, s)	*n.s.*	*n.s.*	*n.s.*	*n.s.*
Vertical activity (3 min)	*n.s.*	*n.s.*	*n.s.*	*n.s.*
Vertical activity (5 min)	*n.s.*	*n.s.*	*n.s.*	*n.s.*
Self-grooming (number)		*n.s.*	*n.s.*	-0.657*
*T-maze test*				
Initial movement (latency of freezing, s)	0.721**	*n.s.*	*n.s.*	*n.s.*
Complete the test (total time, s)	0.751**	*n.s.*	*n.s.*	*n.s.*
*Social Interaction Test*				
Non-social interactions	*n.s.*			
Self-grooming (latency, s)	*n.s.*	-0.716**	*n.s.*	*n.s.*
*Marble interaction test*				
Intact (number of marbles)	*n.s.*	*n.s.*	-0.707**	*n.s.*
Morris water maze				
Day 4 (mean latency, s)	0.684*	*n.s.*	*n.s.*	*n.s.*
***After treatment.***				
*Corner Test*				
Vertical activity (latency, s)	*n.s.*	*n.s.*	0.633**	*n.s.*
Vertical activity (number)	*n.s.*	0.594*	-0.734**	*n.s.*
*Open field test*				
Initial movement (latency of freezing, s)	0.724**	*n.s.*	*n.s.*	*n.s.*
Exist of the center (latency, s)	0.734**	*n.s.*	*n.s.*	*n.s.*
Entrance to the periphery (latency, s)	0.713**	*n.s.*	*n.s.*	*n.s.*
*T-maze test*				
Initial movement (latency of freezing, s)	*n.s.*	*n.s.*	-0.687**	*n.s.*
Complete the test (total time, s)	*n.s.*	-0.653*	*n.s.*	*n.s.*
*Social Interaction Test*				
Social interactions				
Body/face (latency, s)	*n.s.*	-0.599*	*n.s.*	*n.s.*
Vibrating tail (latency, s)	*n.s.*	0.611*	*n.s.*	*n.s.*
Vibrating tail (total time)	*n.s.*	-0.611*	*n.s.*	*n.s.*
Vibrating tail (total no. of episodes)	*n.s.*	0.611*	*n.s.*	*n.s.*

Pearson’s correlations, *P < 0.05, **P < 0.01. n.s., non significant.

### Corner Test (CT)

Increased neophobia exhibited by 3xTg-AD mice before the treatment, as shown by reduced number of corners and rearings and increased latency of rearing, was not ameliorated by risperidone (**Figure 1**). The repeated CT allowed to observe a reduction of the number of corners through the treatment as compared to basal levels, in all groups [**Figure 1A**, NTg (s), *t* = 3.015, gl 11, *P* < 0.05; NTg (r), *t* = 8.517, gl 10, *P* < 0.001; 3xTg-AD(s), *t* = 2,620, gl 11, *P* < 0.05; 3xTg-AD(r), t = 2.776, gl 10, *P* < 0.05]. Likewise, a decrease in vertical activity was observed in NTg (r) mice (*t* = 2.637, gl 10, P < 0.05) and 3xTg-AD (s) mice (*t* = 2.327, gl 11, P < 0.05) (**Figure 1C**). When the four groups were compared, genotype effects were still shown in the ”number of corners visited” in weeks 11 [F(1,43) = 6.623, P < 0.05] and 12 [*F*(1,43) = 15,503, P < 0.001]. The latency of rearing was more sensitive to the genotype effect as it was observed from weeks 9 to 12 [all F(1,43) < 14,450, P < 0.001] and showed a treatment effect in week 9 [F(1,43) = 4.495, P < 0.05] and interaction “genotype × treatment” in week 10 [F(1,43) = 7.151, P < 0.05] (**Figure 1B**). In the variable “number of rearings,” genotype effect was observed from weeks 8 to 12, with all the [F(1,43) < 25.961, P < 0.001] and interaction effect genotype × treatment in week 10 [F(1,43) = 5.975, P < 0.05] (**Figure 1C**).

### Open Field Test (OF)

The delayed ethogram (sequence of behavioral events), the reduced horizontal and vertical activities, and the increased emotionality behavior (urination) exhibited in the OF described an increased anxious-like profile in the 3xTg-AD mice. After treatment, genotype and treatment effects were found as detailed in **Figure 2**. In the NTg mice treated with saline, the latencies of “first movement” (freezing behavior) (t = 2.353, gl 11, P < 0.05) and “grooming” (t = 2.935, gl 11, P < 0.05) were delayed as compared before treatment (**Figures 2A, B**). In addition, there was a decrease in the “total distance traveled” (t = 4.674, gl 11, P < 0.01) (**Figure 2E**). In contrast, the sequence of behaviors [“first movement”, “leaving the center,” “entering into the periphery,” all t(10) < 14,317, P < 0.001; “grooming,” t = 3,681, gl 10, P < 0.01] was faster in those receiving risperidone. A decrease in the “total number of rearings” (t = 2.948, gl 10, P < 0.05) was observed (**Figure 2D**). In the 3xTg-AD mice treated with saline and risperidone, the “latency to enter the periphery” was advanced in time (t = 3.236, gl 11, P < 0.01 and t = 4.071, gl 10, P < 0.01, respectively). Likewise, 3xTg-AD (r) mice showed a decrease in the horizontal activity “distance traveled” (t = 4.104, gl 10, P < 0.01) (**Figure 2E**) and in the vertical activity “total number of rearings” (t = 2.512, gl 10, P <0.05) indicating differences between the untreated phase and the treatment phase at 0.1 mg/kg (**Figure 2D**). When the four groups of treated animals were compared with each other, a genotype effect [F(1,43) = 20.950, P < 0.001], treatment effect [F(1,43) = 6.802, P < 0.05], and interaction effect “genotype × treatment” [F(1,43) = 13.161, P < 0.01] was detected in the variable “total distance traveled.” This was due to the fact that the NTg (r) mice performed greater horizontal exploratory activity with respect to the group of NTg (s) mice, while the 3xTg-AD (r) mice performed less horizontal activity with respect to the 3xTg-AD mice (s) (**Figure 2E**). In addition, in the variables of vertical activity, it was observed that the “latency of the first rearing” showed treatment effect [F(1,43) = 4.376, P < 0.05]. Similarly, the “total number of rearings” indicated genotype effect [F(1,43) = 16.431, P < 0.001], treatment [F(1,43) = 5.594, P <0.001], and interaction “genotype × treatment” [F(1,43) = 4.410, P < 0.05] (**Figures 2C, D**). The variable “grooming latency” showed genotype effect [F(1,43) = 26.950, P < 0.001] because time was delayed in 3xTg-AD mice relative to the other two NTg groups. Regarding the variable “total number of grooming,” a “treatment × genotype” interaction effect was observed [F(1,43) = 8.718, P <0.01] due to a decrease in behavior in the groups of 3xTg-AD mice with respect to the other two NTg groups. In the “urine,” a genotype effect was observed [F(1,43) = 11.133, P < 0.01] due to higher incidence of urine in the 3xTg-AD groups with respect to the NTg groups (**Figure 2F**).

### T-Maze (TM)

Behavioral patterns exhibited in the TM differing between genotypes were referred to emotionality behavior (urination), which was increased in the 3xTg-AD mice. As shown in **Figure 3**, before–after differences were only shown in the groups of 3xTg-AD mice. Animals treated with saline showed an increase in the number of defecation boli (t = -4.486, gl 11, P < 0.01), while the 3xTg-AD (r) group needed longer “time to complete the maze” (t = -2.578, gl 10, P < 0.05). When the four groups of animals were compared with each other, the “latency to cross the intersection” indicated genotype effects [F(1,43) = 5.668, P < 0.05] due to a delay in the 3xTg-AD mice with respect to the two groups of NTg mice. The genotype effect was also evidenced in the variable “exploratory activity” [F(1,43) = 4.496, P < 0.05] because the groups of 3xTg-AD mice performed a greater number of episodes with respect to the two NTg groups. “Genotype × treatment” interaction effects were shown in the “number of fecal boli” [F(1,43) = 4.930, P < 0.05] and “total number of urine” [F(1,43) = 11.381, P < 0.01].

### Social Interaction Test (SIT)

In this test, exhibition of social (**Figure 4**) and non-social (**Figure 5**) behaviors were analyzed. “Face/body contact,” “anogenital contact,” “vibrant tail,” and “aggressive contact” presented distinct latencies of appearance and number of episodes depending on the genotype and were modified by treatment. Before treatment, reduced body/face interaction, horizontal and vertical exploratory behaviors but increased vibrating tail and digging were phenotypic characteristics of 3xTg-AD mice (**Figure 4B**). After treatment, in NTg (s) mice, the face/body contact behavior showed a decrease in the “total number of episodes” (t = 2.217, gl 11, P < 0.05) (**Figure 4C**). In the NTg (r) mice, it was observed that the “face/body” latency was delayed in time (t = 3.006, gl 10, P < 0.05) and the “vibrant tail” latency was advanced (t = 3.738, gl 10, P < 0.01) (**Figure 4A**). In addition, there was a decrease in the contact time “face/body” (t = 4.107, gl 10, P < 0.01) and “anogenital” (t = 3.546, gl 10, P < 0.01) and an increase in “vibrant tail” (t = -4.282, gl 10, P < 0.01) (**Figure 4B**). Likewise, there was a decrease in the number of episodes in “face/body” (t = 5.255, gl 10, P < 0.001), “anogenital” (t = 3.975, gl 10, P <0.01) and an increase in “vibrant tail” (t = -4.282, gl 10, P <0.01) (**Figure 4C**). The mice 3xTg-AD (s) presented a decrease in the duration “anogenital contact” (t = 2.520, gl 11, P < 0.05) (**Figure 4B**) and number of episodes of the “vibrant tail” behavior (t = 2.480, gl 11, P <0.05) (**Figure 4C**). The 3xTg-AD (r) mice showed statistical differences in the appearance of the “face/body contact” behavior (advancement, t = 2.754, gl 10, P < 0.05) and “vibrant tail” (delay, t = -4.643, gl 10, P < 0.01) (**Figure 4A**). These differences were also reflected in the duration and in the number of episodes, since the animals spent more time in the “face/body contact” behavior (t = 2.443, gl 10, P < 0.05) and it increased the number of episodes “face/body” and decreased the number of “vibrant tail” (t = 2.372, gl 10, P < 0.05) (**Figures 4B, C**). When comparing the four groups with each other, the variable “face/body contact latency” showed a “genotype × treatment” interaction effect [F(1,43) = 8.4428, P < 0.01], which was also evidenced in the variable total number of episodes of “face/body contact” [F(1.43) = 9.199, P < 0.01] (**Figures 4A, B**).

In the “latency of anogenital contact,” genotype effects were observed [F(1,43) = 0.818, P < 0.01) with a delay in the groups of 3xTg-AD mice as compared to the other two NTg groups (**Figure 4A**). Conversely, “total anogenital contact time” showed genotype [F(1,43) = 6.766, P < 0.05], treatment [F(1,43) = 5.855, P < 0.05], and interaction “genotype × treatment” [F(1,43) = 4.535, P < 0.05] effects (**Figure 4B**). The genotype effect was also evidenced in the total number of “anogenital contact” episodes [F(1,43) = 9.264, P < 0.01], treatment [F(1,43) = 7.509, P < 0.01], and interaction [F(1,43) = 5.017, P < 0.05] (**Figure 4C**). This was due to the fact that the NTg (s) mice had greater contact duration and number of episodes with respect to the other three experimental groups. In the “tail vibration latency,” “genotype × treatment” interaction effect was observed [F(1,43) = 5.396, P < 0.05] (**Figure 4A**). Likewise, the variable “total time” presented genotype effect [F(1,43) = 7.836, P < 0.01] as well as the variable “number of episodes” [F(1,43) = 6.949, P < 0.05] because groups of 3xTg-AD mice invested more time and presented an increase of episodes with respect to the other two NTg groups (**Figures 4B, C**). In the aggressive contact component, no significant differences were observed in any of the three variables studied for this behavior (**Figures 4A, B, C**).

**Figures 5A and B** illustrate the pattern of non-social behaviors before and after the treatment. In the NTg mice, changes were only observed in those animals treated with the antipsychotic, with a reduced number of rearings (t = 5.519, gl 10, P < 0.001) and visited corners (t = 2.679, gl 10, P < 0.001) as compared to basal levels (**Figure 5B**). In the 3xTg-AD (s) mice, the “latency of rearing” was delayed in time (t = 2.205, gl 11, P < 0.05) resulting in a decrease of the “number of rearings” (t = 3.116, gl 11, P < 0.01). This was also evidenced in the 3xTg-AD (r) mice where the “latency of rearing” was delayed in time (t = -2.323, gl 10, P < 0.05), the “number of rearings” decreased (t = 5.723, gl 10, P < 0.001) and also the “number of visited corners” reduced (t = 3.264, gl 10, P < 0.01) (**Figures 5A, B**). When comparing the four experimental groups with each other, these results were noted as genotype effect in the “latency of the first rearing” [F(1,43) = 24,813, P < 0.001], “total number of rearings” [F(1,43) = 67.857, P < 0.001], and “total number of corners” [F(1,43) = 89.604, P < 0.001] due to the reduced activity of 3xTg-AD mice with respect to the two groups of NTg mice. The treatment effect was observed only in the duration of the burying, which was reduced by risperidone [F(1,43) = 4.185, P < 0.05] in both genotypes. No effects of the genotype or treatment were observed in the “grooming” variable.

### Morris Water Maze (MWM)

**Figure 6** depicts the before and after trial-by-trial (**Figures 6A, B**) and day-by-day (**Figure 6C**) place learning task acquisition curves as well as the platform preference shown in the probe trial to assess memory after treatments (**Figure 6D**). The trial-by-trial performance before treatment indicated that on each day, except for day 3, there was an effect of the “trial” [day 1, F(3,144) = 9.914, P < 0.001; day 2, F(3,144) = 4.082, P < 0.01; day 4, F(3,144) = 7.242, P < 0.001; day 5, F(3,144) = 4.934, P < 0.01]. However, this basal performance in the 3xTg-AD mice resulted in a worse day-by-day acquisition curve with higher latencies as compared to NTg mice, on days 3, 4, and 5. After treatment, statistical significant differences were only shown on day 1, with a “trial × genotype” [F(3,144) = 2.664, P < 0.05] and “trial × genotype × treatment” [F(3,144) = 3.766, P < 0.05] effects. No differences in the acquisition curves were found among the four treated groups. When performance on this first day after treatment was compared to the last performance before treatment, only NTg (s) mice showed worsen performance (t = -2.239, gl 11, P < 0.05). As compared with their respective acquisition curves before treatment, all groups of mice were faster finding the platform in this re-test. NTg (s) showed statistical differences on days 1, 2, and 5 (all t > 2.484, gl 11, P < 0.05), NTg (r) on days 1 and 2 (both t > 1.866, gl 10, P < 0.01), 3xTg-AD (s) from days 1 to 4 (all t > 1.950, gl 11, P < 0.01), and 3xTg-AD (r) on all the days (all t > 2.096, gl 11, P < 0.01).

The second paradigm consisted of the *removal* of the platform. Before treatments, NTg groups showed the highest number of entries in the trained quadrant, where the platform was previously located, with respect to the other quadrants [NTg (s), F(3,47) = 13.48, P < 0.001; NTg (r), F(3,43) = 9.62, P < 0.001]. The number of entries in the trained platform was significantly lower in the 3xTg-AD groups. The detailed analysis showed that preference was also shown for the right quadrant [3xTg-AD (s), F(3,47) = 8.80, P < 0.001; 3xTg-AD (r), F(3,43) = 8.14, P < 0.001] indicating lower focused search strategies in these mice. After treatment, the NTg (s) mice showed an increase in the number of entries in the “trained quadrant” with respect to the other quadrants [F(3,47) = 18.91, P < 0.001]. In contrast, in the other treated groups, an increase in entries was observed in both the quadrant of the platform and the right quadrant [NTg (r), F(3,43) = 31.18, P < 0.001; 3xTg-AD (s), F(3,47) = 18.91, P < 0.001; 3xTg-AD (r) [F(3,43) = 15.27, P < 0.001]. Thus, the variable “number of entries in the right quadrant” indicated “genotype × treatment” interaction effect [F(1,43) = 19.401, P < 0.001]. As compared to their respective performances before treatment, statistical differences were only found in the NTg (r) mice, as a decrease in the number of entries in the opposite (t = 3.203, gl 10, P < 0.01) and left (t = 3.909, gl 10, P < 0.01) quadrant.

### Marble Burying Test (MB)

As illustrated in **Figure 7**, before treatment, 3xTg-AD mice showed an increased pattern of interaction with marbles, with a higher number of marbles that changed position and were buried than NTg counterparts. After treatment NTg (s) mice the “intact” marbles were the least frequent compared to the marbles that changed position and were buried [F(2,35) = 19.51, P < 0.001]. Oppositely, in NTg (r) mice, an increase in the number of marbles “intact” and a decrease in “buried” marbles [F(2,32) = 13.05, P < 0.001] was observed. In the two groups of treated 3xTg-AD mice, no significant changes were observed between the three levels of interaction with marbles. In each group, before and after comparisons indicated differences: NTg (s) mice, in the marble number variables “intact” (t = 5.348, gl 11, P < 0.001) and “buried” (t = -3.824, gl 11, P < 0.001) because after the treatment, there was a decrease in the number of intact marbles and an increase in the number of marbles buried. In the 3xTg-AD (s) mice, an increase in the number of “changed position” marble was observed (*t* = -2.601, gl 11, P < 0.05) and a decrease in “buried” marble (*t* = 3.348, gl 11, P < 0.01). In the groups of animals treated with risperidone NTg (r) and 3xTg-AD (r), no changes were observed. Treatment effects were shown in the “number of intact marble” [F(1,43) = 7.802, P < 0.01] as an increase in its number in the groups treated with risperidone compared to those treated with saline. Conversely, the “number of buried marbles” also indicated treatment effect [F(1,43) = 8.573, P < 0.01] with a reduction as compared to those treated with saline, although this decrease is only significant in the case of NTg (r) animals. Furthermore, in this variable, the interaction effect ”genotype × treatment” was observed [F(1,43) = 6.114, P < 0.05] due to a decrease in the number of marbles buried in the NTg (r) mice and an increase in the 3xTg-AD (r) mice.

### Baseline State of Blood Glucose

Basal state of glucose levels before treatment did not differ between 3xTg-AD and NTg mice (**Figure 8**). However, after treatment, NTg (r) mice showed a marked decrease (t = 7.611, gl 11, P < 0.001). This led to a ”genotype × treatment” effect [F(1,43) = 9.441, P < 0.01].

### Weight

The body weight (BW) of animals was monitored throughout the experimental procedures (**Figure 9**). At the beginning of the experiments, weight of 3xTg-AD mice was higher than that of controls (*t* = -2.213, gl 50, P < 0.05), but differences were attenuated through the experiments. Statistical significance was lost in week 6, just before treatment started (NTg mice, *t* = 4.035, gl 23, P < 0.01; 3xTg-AD mice, *t* = 6.234, gl 27, P < 0.001). During the first week of treatment (risperidone, 0.05 mg/kg), all the groups lost weight [NTg (r) mice, *t* = 2.965, gl 10, P < 0.05; 3xTg-AD (s), *t* = 2.718, gl 11, P < 0.05; 3xTg-AD (r), *t* = 4.633, gl 10, P < 0.01] except NTg mice treated with saline. At 10 weeks of treatment (risperidone, 0.1 mg/kg), the loss of weight was only observable in the 3xTg-AD mice [3xTg-AD (s), *t* = 2.312, gl 11, P < 0.05; 3xTg-AD (r), *t* = 3.478, gl 11, P < 0.01]. The four experimental groups showed reduced “weight immediately after death” as compared to before treatment [NTg (s), *t* = 2.209, gl 11, P < 0.05; NTg (r), *t* = 5.382, gl 10, *P* < 0.0 01; 3xTg-AD (s), *t* = 5.721, gl 11, P < 0.001; 3xTg-AD (r), *t* = 3.937, gl 10, P < 0.01]. When the four groups of animals were compared with each other, no differences were found among them at either of both time points.

### Survival Curve

**Figure 10** illustrates the survival curves and lifespan, which differed among the groups: NTg (s): 1.078 days (36 months), NTg (r): 874 days (28 months), 3xTg-AD (s): 768 days (24 months), 3xTg-AD (r): 660 days (22 months), with a genotype effect in mean lifespan. Lifespan was reduced by AD genotype [F(1,48) = 24.812, P < 0.001] but also by risperidone treatment (shortened 2 months in 3xTg-AD but in 8 months in NTg mice). Survival curves showed that until 14 months of age, all groups exhibited 100% survival. Thereafter, only NTg (s) mice maintained its survival intact until 19 months, that is, 5 months longer than the other groups. When comparing the four groups, their mean survival at 15 months of age showed a genotype [F(1,45) = 4.968, P < 0.05] and “genotype × treatment” [F(1,45) = 4.968, P < 0.05] interaction effects. Thus, although onset of mortality window was 15 months for NTg (r), 3xTg-AD (s), and 3xTg-AD (r), the different slopes in their survival curves indicated different severity levels: NTg (r), survival of 91.61%, 3xTg-AD (s), survival of 85.71%, 3xTg-AD (r), 78.48% (**Figure 11**). Average life expectancy (50% survival) differed among the four groups [F(3,28) = 262.25, P < 0.001]. In NTg mice, the average life expectancy was 24 months, whereas in the two groups of 3xTg-AD mice, it was 18 months. Likewise, when 25% of the survival was analyzed, statistically significant differences with respect to the group of NTg (s) mice [F(3,12) = 137.41, P < 0.001] were found. The Kaplan-Meier test showed significant differences between 3xTg-AD (s) and NTg (s) (Log rank = 7.218, gl 1, P < 0.01) and also among the four experimental groups (Log rank = 22.833, gl 1, *P* < 0.001).

**Table 1** summarizes the meaningful correlation analysis of lifespan with the behavioral phenotype before and after treatment with risperidone for each one of the four experimental groups, while **Figure 12** illustrates the correlations with the whole sample of 46 animals. As shown, in most tests, freezing behavior and activity to complete the task were strongly (P < 0.01) correlated with lifespan in NTg (s). In the NTg (r), it was mostly related to the elicitation of emotionality (grooming), vertical exploratory activity (rearing), and agonistic behaviors (body face, inversely with vibrating tail). Poorest behavioral correlates were found in 3xTg-AD mice, where survival was inversely correlated to a poor marble burying, scarce and slow exploratory activity, and presence of vibrating tail. Survival in 3xTg-AD (r) was negatively correlated with delay in the elicitation of vertical exploratory activity and number of groomings. Correlation with cognitive task was only shown in NTg(s), while the tests and/or variables with predictive value in the whole sample were those related to the BPSD-like phenotype.

## Discussion

The main objective of the present work was to model in 3xTg-AD mice the increased mortality risk induced by the chronic administration of the atypical antipsychotic risperidone shown in patients with AD. This is the first study that considers this objective despite the imperative need to have an animal modeling this vulnerability, an issue that cannot be addressed in clinical studies.

*Experimental design*. First, the battery of behavioral tests allowed us to confirm the AD phenotype of the animals (Giménez-Llort et al., 2007). Thus, we verified the existence of cognitive and BPSD-like behaviors in this initial sample of middle-aged 3xTg-AD mice, an age that has extensively been described to mimic advanced neuropathological stages of the disease (Belfiore et al., 2019). Here it is important to note that although the cognitive deficits in the MWM were confirmed, the inclusion criteria in the “before–after treatment” analysis excluded the animals that died during the period of behavioral assessments. In AD, treatments with atypical antipsychotics are effective in controlling anger, agitation, aggression, and symptoms more typical of the psychotic spectrum such as hallucinations, paranoia, and delusions, while cognitive symptoms, quality of life, and attention do not improve with these treatments (Kalman et al., 2008). In view of the pharmacological action of the antipsychotic treatment, it is therefore interesting to note that the animals before treatment had cognitive deficits but also an increase in the stereotyped behavior of marble burying (usually used to assess the efficacy of antipsychotics) and alterations in social behavior (also modifiable with antipsychotics). Once the phenotype was verified, we evaluated the response of 3xTg-AD mice to chronic treatment with risperidone as compared to the effects exerted on age-matched NTg mice but also as compared to their own phenotype before treatment. Risperidone was used at a dose of 0.1 mg/kg equivalent to those administered in patients with AD and used in most experimental work performed in rodents. As in the case of geriatric patients, the treatment regimen was initiated with a lower dose of 0.05 mg/kg.

In the longitudinal study, the baseline characterization was performed at 12 months of age and the administration of the treatment started at 13 months, which in both cases corresponds to advanced neuropathological stages of the disease. The number of animals used (*n* = 12–14 for each group) was adequate for behavioral studies according to the guidelines on the use of genetically mutated animals and taking into account that at the middle age, the individual variability increases due to age. However, for the study of long-term survival curves, it would have been advisable to use a much greater number, between 40 and 50 animals. This and other works of the literature are limited experimentally to use a reduced number of animals by the difficulty of obtaining the experimental subjects.

*Corner test*. In the modeling of the behavioral effects induced by the atypical antipsychotic risperidone, the behavior of neophobia was evaluated by the CT. At the beginning of the treatment, the 3xTg-AD mice showed a greater behavioral inhibition when confronting novelty, presenting higher levels of neophobia with respect to the NTg mice, in the three variables of the test. Although this response was independent of treatment, it was observed that in NTg (r) mice, the dose of 0.05 mg/kg of risperidone induced a significant increase in vertical activity with respect to the other three experimental groups. In the following week (week 8, dose of 0.1 mg/kg), an attenuation of the differences between genotypes was observed, now only detectable in the rearing variable. In fact, in general, during the rest of the weeks, vertical behavior was the most sensitive to indicate the effects of the factors studied. Repeatedly, the predominant factor was genotype, corroborating the neophobia described in our laboratory in 3xTg-AD mice (Giménez-Llort et al., 2006; Gimenez-Llort et al., 2007; Giménez-Llort et al., 2008 and Giménez-Llort et al., 2010), and in a few cases, treatment effect was observed. Therefore, chronic treatment with risperidone did not modify the neophobic response of the 3xTg-AD mice, and if any effect had, it was sporadically in NTg animals.

*Open-field test*. In agreement with the increased neophobia in the CT, in the OF to assess exploratory activity and anxiety, AD genotype differences were also observed with reduction of vertical and horizontal activity, temporal delay of the grooming behavior, and increased presence of urine. This predominant effect of the genotype factor corroborates the results obtained in the open field in the pretreatment phase and the CTs. Both the corner and open-field tests converged to show the emotional and anxious-like profile of 3xTg-AD mice, in agreement with our previous studies describing the appearance of these behaviors (Giménez-Llort et al., 2007; Giménez-Llort et al., 2008; Giménez-Llort et al., 2010; Giménez-Llort et al., 2018; García-Mesa et al., 2011; García-Mesa et al., 2012) since the early stages to advanced stages of the disease (Giménez-Llort et al., 2006).

The activity variables, both horizontal and vertical, also showed treatment effect *per se* and their effects were influenced by the interaction with the genotype. Thus, most of the effects of risperidone were observed again in NTg (r) animals as an increase with respect to the other three experimental groups. This seems to be a pattern similar to that observed in the CT, but in both horizontal and vertical vectors of locomotor activity, with the NTg (r) group being different from all the others.

When comparing the data at the longitudinal level, it can be seen that in the treatment phase, there is an attenuation of all the responses that could be due to the simple fact of the repetition of the test. Also, it could be attributed to the repeated manipulation or handling of animals. In both cases, these are some of the limitations inherent to the use of behavioral batteries and are enhanced in longitudinal studies and chronic treatments. These limitations are difficult to avoid but can be mitigated by reducing the number of tests and the use of tests with variables with convergent validity, as we have done in our study, and the selection of guidelines for oral administration or subcutaneous pumps.

Risperidone enhanced the behavioral differences but did so differently depending on the genotype. Although the differences between the two groups of NTg animals before the use of drugs may be potentiating this contrast of NTg (r) mice, we have verified that this group, but not the one that would be treated with saline, is the one that shows the most standard values described for the NTg male animals of our colonies. Therefore, in any case, the results of NTg mice treated with saline are downward biased.

About the effect of risperidone, studies with other mouse models for other pathologies, such as the ICR mouse, have found that a low dose of risperidone (0.01 mg/kg, i.p.) decreases the freezing or freezing behavior, and at high doses (0.04 mg/kg, i.p.), spontaneous motor activity is reduced. However, when co-administered with other drugs, it loses effectiveness in its anxiolytic effect (Miyamoto et al., 2004). In male ddY mice of 5 weeks of age, risperidone at a dose of 0.1 mg/kg inhibits methamphetamine-induced hyperlocomotion and at several other doses attenuates that induced by MK-801 through blockade of the 5-HT2A/2C receptor (Uchida et al., 2009). In BTBR mice at doses of 0.125 and 0.25 mg/kg i.p., risperidone decreases exploratory activity (total distance traveled) in the OF (Silverman et al., 2010). The data confirm the dopaminergic action of the drug modulating motor and motivational functions (Rinaldi et al., 2007) and exerting anxiolytic properties.

On the other hand, the literature also suggests that atypical antipsychotics such as risperidone could be useful in attenuating stereotyped behaviors and not just locomotor activity. In the open field, grooming behavior also showed genotype and genotype interaction effects by treatment. Grooming is an activity of daily life linked to hygiene of animals but is also part of copying with stress strategies, and can be developed as a stereotyped behavior that gives and looks easily modified in anxious situations (Kalueff and Tuohimaa, 2004a, Kalueff and Tuohimaa, 2004b). Furthermore, the grooming is used experimentally as a tool to measure behavioral stimulation of D1 receptors (Rimondini et al., 1998) so that risperidone, as regards its antagonist action D1, could induce a decrease in this behavior. In this respect, in the 3xTg-AD mice, it was observed that the grooming behavior appeared later in time with respect to its NTg control, thus corroborating the genotypic differences in the anxious-like profile of these animals described in our laboratory (Giménez-Llort et al., 2006). In these previous studies with old male and female 3xTg-AD mice, the grooming behavior appears later than in the NTg mice and, in turn, aging reduces the duration (Giménez-Llort et al., 2008). Regarding the effect of the treatment, the effects of interaction with the genotype are due to a more frequent grooming behavior in the NTg (r) group of mice, although this selective difference replicates the existing one before treatment. In fact, work on strains of standard mice such as Swiss albino mice indicates that risperidone, at doses of 0.1 and 0.2 mg/kg, decreases the number of grooming (De Oliveira et al., 2008). Similarly, in the BTBR mouse model for autism, they have also observed that at doses of 0.125 and 0.25 mg/kg i.p. they reduce grooming by 40% to 50% with respect to their control, although the reduction in behavior can be confused with levels of sedation (Silverman et al., 2010).

*Social interaction test*. The SIT was found the most complete and sensitive tests to evaluate the deficits of social behavior among 3xTg-AD mice in comparison with those observed among NTg mice of the same age and sex (Torres-Lista and Giménez-Llort, 2019). Here, in the basal characterization before treatment, we replicated the results but, this time, evaluated within a behavioral battery. The 3xTg-AD mice showed results similar to those of the NTg animals in the most common social interaction behavior such as body-face contact. However, the genotype effects indicated less anogenital contact, greater vibrant tail, and absence of aggressiveness in groups of 3xTg-AD mice with respect to what was observed among NTg mice. Besides, the effect of chronic treatment with risperidone was evaluated in these behaviors since atypical antipsychotics are often chosen to treat BPSD symptoms, such as psychosis, aggression, and agitation. Risperidone exerted effects decreasing the anogenital behavior and in the case of the 3xTg-AD mice, it significantly corrected the preponderance of the vibrant behavior recently described as a characteristic of the social interaction pattern of female 3xTg-AD mice (Torres-Lista and Giménez-Llort, 2019). The effects of risperidone interacted with genotype in several variables of social behaviors studied, in such a way that the effects were more intense in the NTg genotype where risperidone decreased social behaviors, bringing them to levels equivalent to those 3xTg-AD groups.

Among the non-social behaviors that appear interspersed during the development of social interactions, the effects of the genotype were observed as a decrease in the horizontal and vertical exploratory activity in the groups of 3xTg-AD mice with respect to the NTg groups. In fact, the behavior of vibrant tail is associated with an immobility of the animal, so that the highest levels of this behavior in the 3xTg-AD mice justify that the exploratory activity, which usually is already lower in these animals, be significantly reduced.

The effects of risperidone were observed only in the variable number of burials, where the drug decreased this behavior, considered mimicking psychotic type stereotypies or obsessive-compulsive type anxiety. The improvement in this variable in both genotypes and the improvement also of the behavior of vibrant tail in the 3xTg-AD mice suggest that risperidone mainly exerted an antipsychotic/anxiolytic management effect, modifying the features of the anxious trait and/or anxious states, in both genotypes, respectively.

While the effects of atypical antipsychotics in exploratory activity and emotionality are discussed by the scientific community as mainly due to their dopaminergic actions, it is considered that the treatment with risperidone could modify the pattern of the social behavior thanks to its profile as a 5-HT2 antagonist, since these serotonergic receptors have been implicated in social interaction behavior (File and Seth, 2003). In a comparative study in APP/London mice, treatment with risperidone decreased its aggressive behavior and did so consistently to that exerted by 8-OH-DPAT and buspirone, two serotonergic agonists (Moechars et al., 1998). In other models for AD, such as the APP23 mice, treatment with risperidone also attenuates the aggressive behavior of animals (Vloeberghs et al., 2008) whereas in psychosis models, the deterioration of social interaction induced by PCP can be improved with anticholinesterase galantamine (0.3 mg/kg) and with 0.1 mg/kg of risperidone (Wang et al., 2007). Risperidone also corrects the aggression induced by social isolation in male ddY mice (Uchida et al., 2009) and the attack behavior in male albino mice, although it is not exempt from side effects at the level of motor behavior (Rodríguez-Arias et al., 1998). In our animal model, we observed spontaneous increase aggressiveness in 3xTg-AD mice^1^. However, in this study, the presence of this behavior in mice was very low or nonexistent, since it is spontaneous aggressiveness and not induced/enhanced by isolation (animals start from a daily social condition). Even so, the aggressiveness observed in the NTg (s) mice is corrected in the group treated with risperidone NTg (r). In the case of 3xTg-AD mice, it is likely that^1^ the behavior of “vibrant tail” acts as a dissipating mechanism that temporarily attenuates the response of directed aggressiveness, with no “aggressiveness” observed.

*T-maze*. The spontaneous alternation in the TM is a paradigm mostly used to evaluate working memory, which also includes aspects such as exploratory activity and emotionality/anxiety. It is a test based on the possibility that the rodent chooses one of the arms arranged in a T-shape. Many brain areas, such as the hippocampus, septum, prefrontal cortex, and the basal forebrain, as well as several neurotransmitters, such as dopamine and norepinephrine, are involved in the implicit working memory involved in the performance of the test (Zhang et al., 2004; Deacon and Rawlins, 2006). Variables such as latency to cross the intersection of the maze allow, in addition, to assess the copying with stress strategies of animals, this being a variable that correlates with a worse neuroimmunoendocrine function, indicators of accelerated aging in mice, and premature death (Guayerbas et al., 2001). In our study, no differences were observed in the number of execution errors, but the efficiency to complete the different phases of the test was diminished in 3xTg-AD mice and slightly affected by risperidone. Thus, in phase 2 of the study, the genotype effects were observed in the emotionality as an increase in the presence of urine with respect to the NTg groups. Later, in phase 3, the variable latency to intersection and time to complete the maze denoted the deficiencies of the 3xTg-AD.

The effects of the treatment were only observed in phase 2, in which the groups treated with risperidone at the dose of 0.05 mg/kg needed more time to complete the exploration of the maze with respect to the groups that received saline. In addition, in the 3xTg-AD (r) mice, the time to complete the maze was also higher compared to that needed before treatment. The effects of the interaction between genotype and treatment were observed in phase 3 at the emotional level in both bowel movements and urine.

In our previous studies, we have observed that the latency to cross the intersection is a variable that reflects changes and subsequent deficiencies in the stress management strategies of animals and is related to the deterioration of the homeostasis of the neuroimmunoendocrine system, due to the age itself and, above all, in the presence of transgenes (Giménez-Llort et al., 2010; Giménez-Llort et al., 2012). In 6-month-old female 3xTg-AD mice, these changes are reflected as a greater speed to reach the intersection of the TM with respect to the controls in a typical flight behavior, while at later ages, the strategy fight-or-flight chosen to combat the acute stressful situation is the petrifaction (Giménez-Llort et al., 2010, Giménez-Llort et al., 2012). These observations agree with those of the reference laboratory that described this functional relationship in a longitudinal study with female OF-1 Swiss mice. Thus, De la Fuente’s laboratory showed that the animals that spend more time in the TM show high levels of emotionality/anxiety and have a less competent immune system with respect to those that explore more quickly. In addition, the animals that showed lower performance in the TM showed a reduction in survival compared to those that obtained better results (Guayerbas et al., 2001).

The decrease of spontaneous alternation in the TM has also been observed in other transgenic models of AD such as Tg2576 (Lalonde et al., 2003) and APP/PS1 (Tempier et al., 2013) mice, in animal models for schizophrenia as STOP-null mice (Delotterie et al., 2010), or pharmacological models by selective blockade of dopamine D1 and D2 receptors in the pre-limbic region of the prefrontal cortex (Rinaldi et al., 2007). The effects of antipsychotics differ depending on the model, the drug, and the duration of treatment. Thus, in APP/PS1 mice, the chronic treatment with Quetiapine for 7 and/or 10 months normalized the anxiety-like behavior observed in the maze, minimized memory deterioration, and decreased Aβ plaques in the brain. In the STOP-null mice, treatment with risperidone only induced a trend to reduce the spontaneous alternation in the Y-maze (Delotterie et al., 2010). Other work in the radial maze has shown beneficial effects of olanzapine and clozapine on the memory impairment of male BALB/c mice, although they were not exempt from some extrapyramidal effects (Mutlu et al., 2012).

Together with the CT and the open field, the results in the TM provided new data to confirm the emotional/anxious-like profile of the 3xTg-AD mice and their worse capacity to cope with stress; however, it did not show problems in working memory as measured by spontaneous alternation. The chronic treatment with risperidone, in any case, worsened this AD-profile and the exploratory efficiency of NTg animals, probably due to the extrapyramidal effects. Still, being an atypical antipsychotic and administered in a low dose regime, it is considered that these side effects are much lower than in other classic antipsychotics.

*Morris water maze*. To evaluate short- and long-term spatial reference learning and memory, the MWM was used (Morris, 1981; Morris, 1984). All groups showed the same acquisition curve in either the “trial-by-trial” or the “day-by-day” analysis for short-term and long-term learning and memory, respectively. Likewise, in the probe trial with removal of the platform, all the groups distinguished the quadrant of the platform with respect to the other quadrants, although the risperidone treatment decreased the selective search of the platform incorporating one of the adjacent quadrants in the preference. The preference of this adjacent area indicates less focused goal-directed swimming strategies (Baeta-Corral and Giménez-Llort, 2015). The interaction between genotype and treatment in the preferences of the two adjacent quadrants indicated that the effects of risperidone affect the two genotypes differently, although in general these and the other data do not indicate deficiencies *per se* but rather changes in search strategies. Thus, as in the case of working memory, risperidone did not affect the results decisively since the animals continued to distinguish what had been the position of the platform. It should also be noted that the parity in the results of this test is surely conditioned by the fact of the previous knowledge of the paradigms since the genotype differences that were observed in the first experience in the water maze no longer exist among the saline groups. The latencies to reach the platform in the first trials were very low, equivalent to the third day of testing before treatment, supporting this interpretation. Here, it is important to remind that the analysis is based on a “before–after design” on censured data, that is, excluding the animals that died during phases 1, 2, and 3. Since the performance of the initial sample of animals was in agreement with the cognitive deficits described in this animal model at 12 months of age, the present results in the before–after analysis suggest a mortality bias, with exclusion of animals with worse life prognostic as determinant to the deficits in the overall group performance. Also, this suggests that the effects of risperidone illustrated here were those exerted in the “less worse” animals, as those that died during the behavioral assessments were excluded.

In some cases, in this mouse model, it has also been observed that the treatments, according to their pharmacological actions, selectively affect different aspects of the learning and memory process. This is the case of the study with 10-month-old 3xTg-AD mice in both sexes, where it was found that the chronic treatment of 5 months with paroxetine improved the deficit of the space navigation in both males and females, without affecting the speed of swimming or the distance traveled, which suggests a conservation of cognitive functions (Nelson et al., 2007). Likewise, studies with 12-month-old female 3xTg-AD mice treated with melatonin found that the treatment improved learning retention from platform position (García-Mesa et al., 2012). In APP/PS1 mice treated with quetiapine, the continuous administration of 4 to 7 months of the antipsychotic decreased the number of plaques of Aβ in the cortex and in the hippocampus of the animals and reduced memory loss, also attenuating the anxiety-like behavior (He et al., 2009). On the other hand, it has been described that olanzapine does not affect the processes of acquisition, consolidation, or recovery in the MWM test (Hou et al., 2006). However, the same work shows that clozapine and haloperidol appeared to affect the acquisition process and consolidation and induced a deterioration in spatial learning (Hou et al., 2006).

In fact, the effects of antipsychotics on learning and memory in processes that occur properly with psychosis are controversial, since there are studies in rats that have indicated that the classic antipsychotic haloperidol and the atypical antipsychotic risperidone, at certain doses, affect cognitive processes (Didriksen et al., 2007), while clozapine and sertindole were effective in the treatment of psychosis without producing detrimental effects on cognition (Mutlu et al., 2012). It is more than probable that these discrepancies in the pharmacological actions are due to the differences they present in the profile of pharmacological selectivity by different neurotransmission systems such as dopamine and serotonin. In this respect, risperidone is classified as a “qualitatively atypical” antipsychotic agent with a relatively low incidence of extrapyramidal effects when given at low doses that have a serotonergic antagonist action higher than dopaminergic.

*Marble burying test*. The burial test for marbles, which is used for the detection of new antidepressants, anxiolytics, and antipsychotics (Njung’e and Handley, 1991; Bruins Slot et al., 2005 and Kaurav et al., 2012), was found sensitive to detect alterations in the 3xTg-AD mice (Torres-Lista et al., 2015). The response patterns were clearly different, since the level of interaction of the NTg mice with the marbles results in half of the objects being changed of position, while the other two quarters remained intact or have been buried. In the 3xTg-AD mice, the burying behavior is enhanced so that more than half of the objects appear buried at the end of the test, a quarter changed position, and only the remaining 10% remained intact.

The marble burying behavior of marbles was studied again to evaluate the effect of the antipsychotic. However, it is interesting to observe previously, in the results of the groups treated with saline, that the behavioral pattern described above for one and another genotype has been significantly modified by the protocol of chronic manipulation. In the NTg mice, the interaction with the objects increased, enhancing the burying of the marbles, which are now quantitatively equivalent to the marbles that changed position. In contrast, in 3xTg-AD mice, chronic saline treatment reduced the interaction with the objects, significantly decreasing the number of marbles buried in favor of the marbles that changed position. Taken together, these effects reduce genotype differences.

When all the factors are evaluated together, the observation of the absence of genotype effect is ratified. Risperidone increased the number of marbles intact and decreased the number of marbles buried with respect to the groups administered with saline, so, in general, risperidone reduced the interaction with objects. These results corroborate those observed in the burying/digging behavior during the SITs. Converging, differences were also found between genotypes with greater burying behavior in the 3xTg-AD mice, the genotype differences disappeared when studied during the treatment, and risperidone decreased burying behavior in the two groups. Although the burying/digging behavior that is evaluated during the SIT is not a behavior with respect to any object, the behavior is similar to that which is measured, in a more directed way, in the marble burying test.

Investigations with other mouse models such as male ICR treated with risperidone at a dose of 1 mg/kg, p.o. have observed that risperidone treatment is effective in reducing the number of buried marbles, although it also reduces locomotor activity (Matsushita et al., 2005). This effect is also shown in male BTBR mice (Gould et al., 2011) and in NIH Swiss male mice (Li et al., 2006). Likewise, in male NMRI mice, risperidone, at a dose of 0.16–0.63 mg/kg, significantly reduces marble behavior and locomotor activity, suggesting that the antagonist action of the drug 5-HT2A receptor may contribute to the effectiveness of the burying of marbles and having anxiolytic effect (Bruins Slot et al., 2005). According to these results, it can be said that risperidone, at a low dose without cataleptic effect, can effectively reduce the number of buried marbles (Bardin et al., 2006).

*Glucose levels*. Antipsychotic drugs can cause a variety of metabolic problems such as weight gain, hyperglycemia, lipid abnormalities, and development of type 2 diabetes. Given these serious health risks, FDA requested that antipsychotics of second generation such as clozapine, olanzapine, risperidone, quetiapine, ziprasidone, and aripiprazole should be labeled to indicate that they increase the risk of developing diabetes (Cope et al., 2009 and Savoy et al., 2010). In the case of increased vulnerability to higher mortality risk in AD induced by atypical antipsychotics, the metabolic syndrome has been hypothesized initially as one of the possible risk factors. Antipsychotics bind with high affinity to a wide variety of neurotransmitter and transporter receptors that could be involved in various metabolic effects; however, the underlying mechanisms are not entirely clear.

In the basal study, we evaluated baseline fasting glucose levels as well as ip glucose tolerance test (IPGTT) and we repeated the measurement of basal glucose levels, this time without fasting, after chronic treatment with risperidone. The glucose tolerance test was ignored because it also involves fasting in animals added to the normal fast in the light phase and the consequent loss of weight that, at these ages, could affect the survival curves of the animals.

As in the basal characterization phase, no genotype differences were observed during the treatment phase but genotype × treatment interaction effects. These effects resulted, however, from a significant decrease in glucose levels in the NTg (r) mice compared to those in the NTg (s) animals or when the glycemic values of the NTg (r) were compared longitudinally with those obtained before treatment. The results also agree with studies carried out in our laboratory with 3xTg-AD animals at 6 months of age in which we observed, in both sexes, that the basal levels of plasma glucose with and without fasting are normal, with the homeostasis of glucose being the one that is compromised in 3xTg-AD mice, although with aging, the genotype differences are lost (Giménez-Llort et al., 2010). Studies in male C57BL/6 mice administered with risperidone showed a slight increase in blood glucose levels, but only at a low dose of the drug (Dwyer and Donohoe, 2003), while other more recent studies, with males FVB/N mice, observed a significant reduction of plasma glucose (-30%) through the induction of insulin release (Savoy et al., 2010).

*Body weight curves*. The results on weight in patients with AD treated with antipsychotics are somewhat contradictory since there are studies that indicate that the administration of risperidone, olanzapine, or quetiapine at low doses is not associated with weight gain (Rondanelli et al., 2006). However, other research indicates that the use of olanzapine and quetiapine significantly increases weight in women and modifies cholesterol levels (Zheng et al., 2009). At the experimental level, weight measurements are a good indicator of the health status of the animal. In addition, in this study, we can evaluate the possible effect of chronic treatment with risperidone in this variable, in addition to the fact that weight is required to administer the correct dose of treatment.

For these reasons, it was decided to make a longitudinal follow-up of the weight from the beginning of the experimental process to the end of the mice’s life. According to our own data, higher BW was found in the 3xTg-AD mice (Giménez-Llort et al., 2007), and as they advanced in age, these differences were attenuated. Risperidone produced a weight loss only in 3xTg-AD mice, while groups of NTg mice maintained their BW. In the final stages of the animal’s life, an acute weight loss was observed and this, immediately after the death, was found to be diminished in the four experimental groups, although the process of cachexia and the dehydration intrinsic to the death process make the data to lose accuracy. Previous studies with 6-month-old 3xTg-AD mice (males and females) have observed a decrease in the weight curve only in the group of females in potentially stressful situations (Giménez-Llort et al., 2010). This could be because they are more sensitive to repeated handling or the exposure of behavioral tests; however, it cannot be stated to what extent weight loss can be associated with emotional reactivity.

Several studies suggest that weight reduction may be associated with decreased activity but depending on the doses administered. Some authors have shown a relationship of the neuroleptic effects of olanzapine, ziprasidone, and risperidone with the decrease in food intake, the reduction of body mass, and motor activity since these changes are only present in wild-type mice but not in D2R knockout mice (Yoon et al., 2010). However, treatment with risperidone 4 mg/kg p.o. in female C57BL/6J mice of 12 weeks of age induces an increase in food consumption, the corresponding weight gain, an increase in body temperature during the light phase, and a reduction in the activity during the dark phase (Cope et al., 2009).

*Life expectancy and survival*. Treatment with atypical antipsychotics may double the risk of mortality in elderly patients with dementia, although this risk is similar to or even lower than antipsychotics. The increase in risk is faster in antipsychotic treatment and does not seem to be related either to the dose or to the type of substance (Vilalta-Franch et al., 2008). Recent studies have indicated that patients with dementia and prescription antipsychotics such as thioridazine, chlorpromazine, haloperidol, trifluoperazine, and risperidone to treat neuropsychiatric symptoms increased the long term risk. The most pronounced difference will be between 24 and 36 months after the start of treatment (Ballard et al., 2009; Kales et al., 2012). In addition, on other mental pathologies such as bipolar disorder in the elderly patient, risperidone has a higher incidence of mortality associated as compared to quetiapine (Bhalerao et al., 2012).

Our longitudinal study replicates the increased mortality described in male 3xTg-AD mice as compared to age-matched NTg mice by our laboratory (Giménez-Llort et al., 2008) and demonstrates that this animal can model the vulnerability to increased mortality risk associated with risperidone in the human patients. Thus, the group of NTg (s) mice maintained their survival intact until 19 months of age, while the start of mortality was during 15 months of age for the groups of NTg (r), 3xTg-AD (s), and 3xTg-AD (r). In previous studies with 15-month-old males 3xTg-AD and NTg mice, the increase in mortality, evaluated from 6 to 15 months of age, was 40%. In the present study, at the same age, the percentage was 15% since survival was recorded from 12 months and, therefore, mortalities that occurred earlier (i.e., from 6 months of age) were not counted (Giménez-Llort et al., 2008). Increased mortality in 10–20-month-old male 3xTg-AD mice as compared to B6129F2 wild-type controls has also been found by other laboratories and shown to be accompanied by elevated frailty scores (Kane et al., 2018).

The total life expectancy, the mean life expectancy, and the life expectancy with a lifespan of 25% showed the predominant effects of the genotype, but also the effect of risperidone in the range of ages below. Therefore, although the survival curves in the mice treated with risperidone and saline showed similar slopes in the four experimental groups, the initial vulnerability and from the mean life expectancy determined important changes in the maximum survival recorded. In NTg mice, this maximum was reduced by 8 months with the risperidone treatment (from 36 to 28 months), and in groups of 3xTg-AD mice, the maximum survival was reduced by 2 months (from 24 to 22 months). The data suggest, in addition, that risperidone exerted deleterious effects in NTg animals that equaled them in vulnerability to animals with 3xTg-AD genotype. This deleterious effect of risperidone in NTg animals was mainly observable between 15 and 17 months of age, although it reappeared in the age ranges corresponding to their half-life expectancy (24 and 25 months, respectively) and the difference persisted until the end of the curve.

The behavioral correlates for survival pointed out variables and tests related to the BPSD-like phenotype, in agreement with the neuroimmunoendocrine hypothesis (Giménez-Llort et al., 2014) pointing at sex-specific neuroimmunoendocrine aging in 3xTg-AD mice and its relation with longevity (Giménez-Llort et al., 2008). The crosstalk refers to the oxi-inflamaging theory developed in PAM and NPAM mice where the divergence of the speed of the aging process (premature in PAM and normal in NPAM) was correlated with immune function, oxidative stress, and worse/better copying with stress strategies in the TM (Guayerbas et al., 2001). While neophobia and worse long-term memory were found to be correlated with reduced survival in female NTg and 3xTg-AD mice (Torres-Lista et al., 2017), it was surprising that memory variables did not correlate with survival in the present work, probably due to the mortality bias, as discussed above. Thus, this was probably because the impairment was less pronounced in the current male sample, as correlation was only found with the level of optimization of the performance (day 4) in the water maze. Interestingly, the effects of risperidone on social behaviors in the NTg mice were the ones to correlate with survival, with faster body/face interaction and delayed elicitation of vibrating tail being correlated with longer longevity of NTg (r) animals, but also when the whole sample of animals was considered. This is important to note, since, to our knowledge, it is the first time that social behavior is being related to lifespan.

Risperidone is mainly metabolized in the liver (Mannens et al., 1993 and Zhou et al., 2006) by the enzyme cytochrome P-450 2D6 (CYP2D6), which has more than 20 genetic polymorphisms (Leysen et al., 1988; Mannens et al., 1993 and Matsubara et al., 2007). Whether biological aspects related to the individual variability in drug metabolism either by polymorphisms or by hepatomegaly (Marchese et al., 2014) explain the increased mortality risk remains to be elucidated.

In summary, in this longitudinal study, the effects of the chronic administration of risperidone on cognition and BPSD-like (motor, NPS, emotional, and social) symptoms were evaluated in male 3xTg-AD and NTg mice, as well as the short- and long-term impact on their survival. It was observed that the factors “genotype,” “treatment,” and “genotype × treatment” interaction effects were present in most of the behaviors studied, such as decreased neophobia, and improved the development of exploratory activity in the open field in NTg mice but had a weak effect on these variables in the 3xTg-AD mice model. Risperidone was effective in reducing the number of marbles buried in the groups of mice that received antipsychotics with respect to the groups that were given saline. The study replicated, in 3xTg-AD mice, the increased mortality risk associated to risperidone observed at a clinical level in humans. Thus, despite the fact that risperidone allowed to modify the alterations shown by the 3xTg-AD mice in their interaction with congeners (social behavior) or environment (burying behavior of marbles), it exerted negative effects by reducing the vertical and horizontal exploratory activity in the tests. What is more relevant, chronic treatment with risperidone severely compromised the life expectancy of the animals already since the beginning of treatment as shown by early mortality windows. The impact of this mortality on the analysis of “before–after treatment” effects on the censured data is relevant to note since it further suggests that the reported effects are those exerted in the “less worse” animals, that is, those that are able to survive during the period of behavioral assessment. In the same way, risperidone exerted especially severe effects in the old NTg animals, also modeling, at the experimental level, the window of early vulnerability described for the adverse effects of cerebrovascular risk in the elderly treated with atypical antipsychotics. The results support the awareness on the use of risperidone and the associated increased risk of mortality in AD, which suggests the relevance of the dosage (dose and treatment period) and that this treatment has to be used in short-term schedules to address the symptoms that cause morbidity and pain in the patient and to diminish the potential of self-hurt. The decision to use atypical antipsychotics should be based on the patient’s medical history and both the benefit and the risk of treatment. At the translational level, the 3xTg-AD mice model and their NTg counterparts can be useful to delimitate critical time windows and for studying the physio-pathogenic factors and underlying causal events involved in this topic of considerable public health significance.

## Data Availability

The raw data supporting the conclusions of this manuscript will be made available by the authors, without undue reservation, to any qualified researcher.

## Ethics Statement

The animal study was reviewed and approved by the protocol CEEAH 2481/ DMAH 8700 entitled “Risk factors and preventive/therapeutical strategies in Alzheimer’s disease: studies in triple-transgenic 3xTg-AD mice” and was approved by Departament de Medi Ambient i Habitatge, Generalitat de Catalunya.

## Author Contributions

Development of the concept and study design: LG-L. Financial resources: LG-L and SL-P. Treatment: LG-L and VT-L. Behavioral studies and data collection: VT-L. Data analysis: VT-L. Data interpretation: VT-L and LG-L. Scientific discussions: VT-L, SL-P, LG-L. Drafting manuscript: VT-L and LG-L. Critical revision of manuscript: VT-L, SL-P, LG-L. Approving final version of manuscript: VT-L, SL-P and LG-L.

## Funding

The work received support from Instituto de Salud Carlos III, ISC3 PI10/00283, Spain; Research Agreement LG-L UAB and SL-P, UVaMiD, 2017-SGR-1468 and UAB2019-GE260408. VT-L received a predoctoral grant Fundació La Marató de TV3 2010/062930.

## Conflict of Interest Statement

The authors declare that the research was conducted in the absence of any commercial or financial relationships that could be construed as a potential conflict of interest.

## Footnotes

1 Ramírez-Boix, P., and Giménez-Llort, L. (submitted). Modeling BPSD in rodents: relevance of spontaneous aggressiveness and chronic mild social stress.

## Supplementary Material

Table S1 Genotype effects in the behavioral phenotype before treatment. Statistics: Student t-test, 3xTg-AD vs. NTg (*n*=46), *p<.05, **p<.01, ***p<0.001.
