# Supplementary material for: Impact of Chronic Risperidone Use on Behavior and Survival of 3xTg-AD Mice Model of Alzheimer’s Disease and Mice With Normal Aging
Source: Front Pharmacol. 2019 Sep 24;10:1061. doi: 10.3389/fphar.2019.01061 (PMC6771277; doi:10.3389/fphar.2019.01061)
Supplement: Table S1 — Genotype effects in the behavioral phenotype before treatment. Statistics: Student t-test, 3xTg-AD vs. NTg (n=46), *p<.05, **p<.01, ***p<0.001. [file Table_1.docx]

| **Behavioral response**  **Before treatment** | **Statistics**  **Genotype effects 3xTg-AD *vs*. NTg (*n*=46)** | | | | |  | | | |
| --- | --- | --- | --- | --- | --- | --- | --- | --- | --- |
|  |  |  |  |  | |  | | | |
| **Corner Test** |  |  |  |  | |  | | | |
| Vertical activity (latency, s) | F_(1,45)_= 5.367 | P<0.01 | * |  | |  |  |  |  |
| Vertical activity (*n*) | F_(1,45)_= 8.110 | P<0.001 | *** |  | |  |  |  |  |
| Horizontal activity (*n*) | F_(1,45)_= 25.678 | P<0.001 | *** |  | |  |  |  |  |
| **Open field test** |  |  |  |  | |  |  |  |  |
| Initial freezing (latency, s) | F_(1,45)_= 0.067 | P=0.977 |  |  | |  |  |  |  |
| Exit of the center (latency, s) | F_(1,45)_= 0.424 | P=0.737 |  |  | |  |  |  |  |
| Entry into the periphery (latency, s) | F_(1,45)_= 1.879 | P=0.148 |  |  | |  |  |  |  |
| Vertical activity (latency, s) | F_(1,45)_= 1.367 | P=0.266 |  |  | |  |  |  |  |
| Self-grooming (latency, s) | F_(1,45)_= 2.766 | P=0.054 |  |  | |  |  |  |  |
| Self-grooming (total *n*) | F_(1,45)_= 11.455 | P<0.001 | *** |  | |  |  |  |  |
| Vertical activity (1 min) | F_(1,45)_= 7.917 | P<0.001 | *** |  | |  |  |  |  |
| Vertical activity (2 min) | F_(1,45)_= 3.526 | P<0.05 | * |  | |  |  |  |  |
| Vertical activity (3 min) | F_(1,45)_= 4.516 | P<0.01 | ** |  | |  |  |  |  |
| Vertical activity (4 min) | F_(1,45)_= 4.774 | P<0.01 | ** |  | |  |  |  |  |
| Vertical activity (5 min) | F_(1,45)_= 2.567 | P=0.067 |  |  | |  | | |  |
| Vertical activity (total *n*) | F_(1,45)_= 7.809 | P<0.001 | *** |  | |  | | |  |
| Horizontal activity (total distance, cm) | F_(1,45)_= 5.122 | P<0.01 | ** |  | |  | | |  |
| Defecation (total *n* of episodes) | F_(1,45)_= 0.466 | P=0.707 |  |  | |  | | |  |
| Urine (total *n* of episodes) | F_(1,45)_= 5.602 | P<0.01 | ** |  | |  | | |  |
| **Social Interaction Test** |  |  |  |  | |  | | |  |
| **Social interactions** |  |  |  |  | |  | | |  |
| Body/face (Latency, s) | F_(1,45)_= 6.348 | P<0.01 | ** |  | |  | | |  |
| Body/face (total time, s) | F_(1,45)_= 13.689 | P<0.001 | *** |  | |  | | |  |
| Body/face (total *n* of episodes) | F_(1,45)_= 20.580 | P<0.001 | *** |  | |  | | |  |
| Vibrating tail (Latency, s) | F_(1,45)_= 19.443 | P<0.001 | *** |  | |  | | |  |
| Vibrating tail (total *n* of episodes) | F_(1,45)_= 8.611 | P<0.001 | *** |  | |  | | |  |
| Vibrating tail (total time, s) | F_(1,45)_= 8.536 | P<0.001 | *** |  | |  | | |  |
| Ano-genital (Latency, s) | F_(1,45)_= 2.636 | P=0.062 |  |  | |  | | |  |
| Ano-genital (total number of episodes) | F_(1,45)_= 2.273 | P=0.094 |  |  | |  | | |  |
| Ano-genital (total time, s) | F_(1,45)_= 2.368 | P=0.084 |  |  | |  | | |  |
| Aggressiveness (Latency, s) | F_(1,45)_= 0.941 | P=0.430 |  |  | |  | | |  |
| Aggressiveness (total number of episodes) | F_(1,45)_= 0.941 | P=0.430 |  |  | |  | | |  |
| Aggressiveness (total time, s) | F_(1,45)_= 0.941 | P=0.430 |  |  | |  | | |  |
| **Non-social interactions** |  |  |  |  | |  | | |  |
| Corner (total *n* of episodes) | F_(1,45)_= 7.064 | P<0.01 | **** |  | |  | | |  |
| Rearing (Latency, s) | F_(1,45)_= 1.592 | P=0.206 |  |  | |  | | |  |
| Rearing (total *n* of episodes) | F_(1,45)_= 15.871 | P<0.001 | ***** |  | |  | | |  |
| Digging (Latency, s) | F_(1,45)_= 3.068 | P<0.05 | *** |  | |  | | |  |
| Digging (total *n* of episodes) | F_(1,45)_= 1.805 | P=0.161 |  |  | |  | | |  |
| Self-grooming (Latency, s) | F_(1,45)_= 1.611 | P=0.201 |  |  | |  | | |  |
| Self-grooming (total *n* of episodes) | F_(1,45)_= 1.124 | P=0.350 |  |  | |  | | |  |
| **T-maze test** |  |  |  |  | |  | | |  |
| Initial movement (latency of freezing, s) | F_(1,45)_= 0.448 | P=0.720 |  |  | |  | | |  |
| Reach criteria (s) | F_(1,45)_= 1.145 | P=0.342 |  |  | |  | | |  |
| Complete the test (total time) | F_(1,45)_= 0.330 | P=0.804 |  | |  | |  |  |  |
| Defecation (total *n* of episodes) | F_(1,45)_= 0.200 | P=0.896 |  | |  | | |  |  |
| Urine (total *n* of episodes) | F_(1,45)_= 1.247 | P=0.305 |  | |  | | |  |  |
|  |  |  |  | |  | | |  |  |
| **Morris water maze** |  |  |  | |  | | |  |  |
| **Day-by-day**  Day 1 (Mean latency, s) | F_(1,45)_= 1.448 | P=0.242 |  | |  | | |  |  |
| Day 2 (Mean latency, s) | F_(1,45)_= 0.146 | P=0.932 |  | |  | | |  |  |
| Day 3 (Mean latency, s) | F_(1,45)_= 2.145 | P=0.109 |  | |  | | |  |  |
| Day 4 (Mean latency, s) | F_(1,45)_= 2.019 | P=0.126 |  | |  | | |  |  |
|  |  |  |  | |  | | |  |  |
| Day 5 (Mean latency, s) | F_(1,45)_= 1.302 | P=0.287 |  | |  | | |  |  |
| **Trial-by-trial**  PT11 (Latency, s) | F_(1,45)_= 2.919 | P<0.05 | *** | |  | | |  |  |
| PT12 (Latency, s) | F_(1,45)_= 2.250 | P=0.096 |  | |  | | |  |  |
| PT13 (Latency, s) | F_(1,45)_= 0.293 | P=0.830 |  | |  | | |  |  |
| PT14 (Latency, s) | F_(1,45)_= 0.287 | P=0.835 |  | |  | | |  |  |
| PT21 (Latency, s) | F_(1,45)_= 1.457 | P=0.240 |  | |  | | |  |  |
| PT22 (Latency, s) | F_(1,45)_= 0.721 | P=0.545 |  | |  | | |  |  |
| PT23 (Latency, s) | F_(1,45)_= 1.229 | P=0.311 |  | |  | | |  |  |
| PT24 (Latency, s) | F_(1,45)_= 0.038 | P=0.990 |  | |  | | |  |  |
| PT31 (Latency, s) | F_(1,45)_= 0.161 | P=0.922 |  | |  | | |  |  |
| PT32 (Latency, s) | F_(1,45)_= 0.878 | P=0.460 |  | |  | | |  |  |
| PT33 (Latency, s) | F_(1,45)_= 3.447 | P<0.05 | *** | |  | | |  |  |
| PT34 (Latency, s) | F_(1,45)_= 1.140 | P=0.344 |  | |  | | |  |  |
| PT41 (Latency, s) | F_(1,45)_= 2.172 | P=0.106 |  | |  | | |  |  |
| PT42 (Latency, s) | F_(1,45)_= 0.703 | P=0.555 |  | |  | | |  |  |
| PT43 (Latency, s) | F_(1,45)_= 0.649 | P=0.588 |  | |  | | |  |  |
| PT44 (Latency, s) | F_(1,45)_= 1.123 | P=0.351 |  | |  | | |  |  |
| PT51 (Latency, s) | F_(1,45)_= 0.406 | P=0.750 |  | |  | | |  |  |
| PT52 (Latency, s) | F_(1,45)_= 0.793 | P=0.504 |  | |  | | |  |  |
| PT53 (Latency, s) | F_(1,45)_= 0.748 | P=0.530 |  | |  | | |  |  |
| PT54 (Latency, s) | F_(1,45)_= 1.700 | P=0.182 |  | |  | | |  |  |
| **Quadrant preference**  Platform quadrant (*n* of entries) | F_(1,45)_= 1.503 | P=0.228 |  | |  | | |  |  |
| Right quadrant (n of entries) | F_(1,45)_= 6.989 | P<0.01 | **** | |  | | |  |  |
| Opposite quadrant (*n* of entries) | F_(1,45)_= 0.471 | P=0.704 |  | |  | | |  |  |
| Left quadrant (*n* of entries) | F_(1,45)_= 1.303 | P=0.286 |  | |  | | |  |  |
| **Marble interaction test** |  |  |  | |  | | |  |  |
| Intact (*n*) | F_(1,45)_= 2.408 | P=0.079 |  | |  | | |  |  |
| Change of position (*n*) | F_(1,45)_= 6.822 | P<0.01 | **** | |  | | |  |  |
| Buried (*n*) | F_(1,45)_= 3.748 | P<0.05 | *** | |  | | |  |  |
| **IPGTT** |  |  |  | |  | | |  |  |
| Glucose concentration mg/dl | F_(1,45)_= 0.452 | P=0.717 |  |  | |  | | | |
|  |  |  |  |  | |  | | | |
| **Weight (g)** |  |  |  |  | |  | | | |
| Week 1 | F_(1,45)_= 5.803 | P<0.05 | * |  | |  | | | |
| Week 6 | F_(1,45)_= 4.126 | P<0.05 | * |  | |  | | | |
